# Development of a disease-specific quality of life questionnaire for patients with aplastic anemia and/or paroxysmal nocturnal hemoglobinuria (QLQ-AA/PNH)—report on phases I and II

**DOI:** 10.1007/s00277-016-2867-8

**Published:** 2016-11-11

**Authors:** Martha Groth, Susanne Singer, Cathrin Niedeggen, Andrea Petermann-Meyer, Alexander Röth, Hubert Schrezenmeier, Britta Höchsmann, Tim H. Brümmendorf, Jens Panse

**Affiliations:** 1 Department of Oncology, Hematology, Hemostaseology and Stem Cell Transplantation, Medical Faculty, University Hospital RWTH Aachen, Pauwelsstr. 30, 52074 Aachen, Germany; 2 Division of Epidemiology and Health Services Research, Institute of Medical Biostatistics, Epidemiology and Informatics, University Medical Centre, Mainz, Germany; 3 Department of Hematology, University Hospital Essen, University of Duisburg-Essen, Essen, Germany; 4 Institute of Clinical Transfusion Medicine and Immunogenetics, German Red Cross Blood Transfusion Service and Institute of Transfusion Medicine, University of Ulm, Ulm, Germany

**Keywords:** Aplastic anemia, Paroxysmal nocturnal hemoglobinuria, Quality of life, Bone marrow failure syndromes

## Abstract

Acquired aplastic anemia (AA) and paroxysmal nocturnal hemoglobinuria (PNH) are interrelated ultra-rare diseases. Quality of life (QoL) evaluation tools used in studies for AA and PNH are unspecific and designed for cancer patients (e.g., the European Organization for Research and Treatment of Cancer Quality of Life Questionnaire, EORTC QLQ-C30). Given the complexity of AA and PNH, variation in symptoms and treatments, younger age of many patients, and the fact that AA and PNH are not classified as malignant diseases, it is likely that cancer-specific questionnaires are inappropriate. We generate an AA/PNH-specific QoL questionnaire (QLQ-AA/PNH), performed according to EORTC guidelines. QoL issues were obtained from the literature and interviews with patients and physicians (phase I), then ranked by patients and physicians. In phase II, items were created. Patients in more than 25 German and Swiss cities were interviewed face to face. In phase I, interviews of 19 patients and 8 physicians specialized in AA/PNH treatment resulted in 649 QoL issues; these were condensed to 175 and graded according to their importance by 30 patients and 14 physicians (phase II). Five physicians took part in phases I and II. Altogether, 97 issues were rated important. Twelve EORTC QLQ-C30 items were not rated important, while several new QoL aspects were brought up. Modifications in wording and phrasing led to two questionnaires with 77 items regarding general QoL aspects and 20 items regarding medical care. Important QoL aspects of PNH/AA patients are inappropriately captured with available QoL tools. Developing a new QoL questionnaire specific for this patient group is warranted.

**Electronic supplementary material:**

The online version of this article (doi:10.1007/s00277-016-2867-8) contains supplementary material, which is available to authorized users.

## Introduction

Both acquired aplastic anemia (AA) and paroxysmal nocturnal hemoglobinuria (PNH) are ultra-rare diseases with a yearly incidence within the western hemisphere of 1.3 to 2 per million [1, 2]. The number of patients newly developing AA, PNH, or overlap syndromes, e.g., within Germany, is estimated to be 250 per year; however, not all of them are properly diagnosed so that the actual number of newly diagnosed patients is somewhat lower. Age distribution for AA shows a bimodal distribution with a peak in young adults, while the mean age at diagnosis of PNH is between 30 and 45 years. PNH and AA are interrelated diseases and cannot be viewed separately as both belong to the group of bone marrow failure syndromes (BMFS) [3, 4].

For patients with PNH, the median overall survival before the introduction of the complement inhibitor eculizumab was between 10 and 22 years with about a third of patients dying within the first 10 years after diagnosis [5–8]; whereas now, basically all patients survive the first year and data from the long-term safety and efficacy of Eculizumab in 195 patients with hemolytic PNH show that consequent complement inhibitor treatment results in a 3-year survival estimate of 97.6%, i.e. as in age-matched controls [9]. Allogeneic bone marrow transplantation (BMT), rarely used in patients with PNH, is the treatment of choice for patients below the age of 40 with severe AA and an available HLA-identical sibling [2]. Treatment decisions in AA for patients without an HLA-identical sibling, older patients, or patients not responding to first-line immunosuppressive therapy (IST) are more complex and involve further courses of IST, BMT from alternative donor sources, or experimental treatment approaches [2, 10].

While considerable research has been performed and published with regard to the pathophysiology and treatment of AA and PNH, much less is known regarding both diseases’ psychosocial issues and their impact on quality of life (QoL).

So far, the only reports on QoL for PNH stem from the original eculizumab trials (TRIUMPH and SHEPHERD) [11, 12] using the European Organization for Research and Treatment of Cancer Quality of Life Questionnaire (EORTC QLQ)-C30 [13] including 87 patients in the TRIUMPH and 93 patients in the SHEPHERD trial. In addition to QoL, fatigue as a major clinical symptom of patients with PNH was prospectively evaluated in both trials using the Functional Assessment of Chronic Illness Therapy Fatigue Instrument (FACIT-Fatigue). Both trials demonstrated that, compared to placebo, treatment with eculizumab resulted in clinically significant improvements in regard to QoL and fatigue.

In another small series with 29 patients, fatigue and abdominal pain were reported as relevant QoL issues [14].

Both questionnaires, the EORTC QLQ-C30 and the FACIT-Fatigue are now routinely used within the PNH registry, a prospectively documenting international registry that has been established following a post-marketing commitment of the eculizumab manufacturer Alexion Pharmaceuticals Inc., requested by both the EMA (European Medicines Agency) and the FDA (Food and Drug Administration) [15].

In AA, QoL assessment is even less standardized and research so far mainly investigated sequelae from bone marrow transplantation [16–18]. Even large studies on treatment outcome mainly used surrogate parameters such as hemoglobin levels and other blood counts or survival to investigate patient burden, but QoL was not measured with validated self-assessed instruments by patients [19–21]. Few studies evaluated QoL aspects in non-transplanted AA patients [22, 23], and there is only one report comparing QoL aspects of patients after BMT to those receiving immunosuppressive therapy retrospectively [24]. However, the QoL evaluation tools used in all these studies were again either surrogate parameters such as treatment toxicity, transfusions and drug requirements, hematologic counts or rates of employment after long-time follow-up or were unspecific for AA patients and transferred from evaluations in cancer patients, e.g., by using the quality-adjusted time without symptoms and toxicity (Q-TWiST) methodology.

Given the complexity of AA and PNH, the variation in symptoms and different treatment approaches (IST, BMT, complement inhibition with eculizumab and others and eventually sequences of all in some patients), the young age of the patients, and the fact that marrow failure syndromes are not classified as malignant diseases, it is likely that the cancer-specific tools used so far are inappropriate to adequately assess the QoL and illness intrusiveness in this group of patients.

Only few experts in the field of PNH can be found and even within the hematologic community, PNH and AA might be underestimated in regard to morbidity and mortality, leading to inadequate or delay of treatment.

We hypothesize that lack of adequate information and counseling, lack of support systems, and the fragmentation of services as well as the varying quality of medical services lead to a profound impact on psychosocial well-being of patients with AA/PNH. Physicians probably often underestimate the resulting disease burden and impacts on QoL.

Based on these considerations, we therefore initiated the development of a AA/PNH-specific QoL instrument (QLQ-AA/PNH) according to EORTC guidelines [25]. The objectives of this study were to identify symptoms and QoL issues reported by patients with AA and/or PNH and identified by physicians working as specialists in the field of AA/PNH treatment and to determine how both groups rate their importance. According to the EORTC quality of life group (EORTC QLG), an issue is a term used to describe and identify a QoL domain that is potentially affected by a disease (malignancy) and/or its treatment [26].

## Methods

### Phase I (generation of issues)

#### Literature review

From March 2012 to May 2012, a systematic literature search was performed using the Medline database and the Website of the American Society of Hematology (ASH) to search the abstracts of the annual ASH meeting. Search criteria included the following terms: aplastic anemia, aplastic syndrome, paroxysmal nocturnal hemoglobinuria, plus one of the following search terms: quality of life, therapy, bone marrow transplantations, immunosuppression, antithymocyte globulin (ATG), eculizumab, patient perspective, symptoms.

Medline search was restricted to articles published between 1985 and 2012, and abstracts from the annual ASH meeting were restricted to be published between 2004 and 2012.

All publications and abstracts were carefully searched for QoL issues mentioned and QoL instruments used.

#### Interviews of patients and physicians specialized in treatment of PNH/AA

Because of the rarity of the disease, special efforts were made to include patients with different disease stages, educational background, and geographical region.

Patients for phase I interviews were approached mainly via physicians and via patient advocacy groups. The internet forum www.pnh-aa.info, founded as a self-help initiative, provides a bulletin board for registered members with links to reference centers and specialists and downloadable information brochures as well as the registered association Aplastische Anämie e.V. (www.aplastische-anaemie.de), which is a patient advocacy group and part of the German Leukemia and Lymphoma Support Group (DLH). Both self-help groups announced our research projects on their website and encouraged patients to take part.

Physicians known as specialists for treatment of AA/PNH, e.g., actively involved in the development of treatment guidelines within Germany, Austria, and Switzerland, were contacted by two of the authors (THB and JP) and patients treated within centers were approached by these physicians.

Additional patients were enrolled during two patient conferences, through a Facebook website of one of the patients and by word-of-mouth from other patients.

Ethical approval was obtained from the ethical committee of the Medical Faculty RWTH Aachen University, and an informed consent letter, which was signed by each participating patient, was phrased in consultation with the data safety manager of the University Hospital RWTH Aachen.

After patients sent back their signed informed consent, one of the authors (MG) contacted them and interviews were appointed. Most patients wanted to be interviewed at home alone; however, two interviews were done in pairs and one group of patients (*n* = 4) was interviewed together in the form of a focus group.

The interviews were open and qualitative. At the beginning of each interview, patients were asked to give a brief overview of their disease history including first symptoms, time to first physician contact, time to diagnosis, and treatment history. Patients were subgrouped into those with predominant AA features, predominant PNH features, and those with overlapping PNH/AA features. In addition, patients were asked to give details regarding their supportive care needs (e.g., information, support by medical staff, psychosocial counseling, and patient support groups) and potential problems experienced due to the non-specificity of symptoms and the local healthcare provision (e.g., delay in diagnosis, lack of local availability of disease-specific experts, appreciation of QoL problems by healthcare professionals).

Patients were invited to report about the diseases’ impact on their quality of life without restricting themselves. All interviews were audio-recorded and subsequently transcribed verbatim. During the focus group interview, issues were recorded on a flip chart by the investigator (MG) and discussed within the group. The session was videotaped and subsequently transcribed in order not to miss any potential issue that was raised. Two reviewers then independently analyzed the transcripts to identify QoL issues mentioned by the patients. Identified issues were then compared and kept if both reviewers identified them as potentially affecting QoL.

Physician interviews were performed accordingly. With the exception of one interview, which was done by telephone, all were done in person by one author (MG). Issues raised by the physicians were then compared to the already collected issues derived from patient interviews and new issues were added to the compilation.

Issues were sorted according to their content into separate categories: physical, psychological, social, financial, healthcare/therapy related. Three reviewers (MG, SS, JP) separately evaluated each issue and rated them as follows: “keep” (keep issue as is), “change” (keep issue, but rephrase), “delete” (delete issue). Issues rated “keep” or “change” from ≥2 reviewers were kept, whereas issues rated “delete” from all three reviewers were deleted without further discussion. Issues rated “keep” or “change” in addition two times “delete” were kept if the reviewer who had performed the interviews rated them relevant after discussion with the other two reviewers.

### Phase II (rating of issues)

#### Review and rating of issues

From the interviews, a list of QoL issues was generated according to [25]. Within a semistructured interview, patients—different from those in phase I—and physicians—five also being interviewed in phase I—were asked to prioritize the 25 most salient issues and rate all issues on a Likert scale (very important (4 points), important (3 points), moderately important (2 points), unimportant (1 point)).

Issues were considered relevant with a high priority for inclusion if ≥2 of the following four criteria were fulfilled: The mean rating of an issue by the total patient sample was ≥2.0; the mean rating of a patient subgroup (AA, PNH/AA, or PNH) was ≥2.5; ≥20% of patients ranked an issue among the top 25 issues; ≥ 30% of physicians ranked an issue among the top 25 issues. Patients and physicians were also asked if any issues were missing. In contrast to EORTC guidelines, patients and physicians were asked to rate issues as being important or not considering the whole course of their disease and not just the last couple of weeks. This was done because PNH and AA patients often experience their diseases as a chronic, long-lasting state with various “disease flares” and patients after bone marrow transplantation might experience completely different disease features and treatment-related morbidities before versus after the transplantation procedure.

#### Generation of items

All issues fulfilling the abovementioned criteria were reworded into items so that they can be completed with the same response categories as it is required for a self-administered questionnaire. The wording was suggested by one author (MG) and reviewed by two authors (SS, JP) until a consensus was reached.

All interviews, recordings, transcriptions, and generation of issues and items were done in German. For the purpose of publication this manuscript translation of issues and items into English language was done by one author (JP) with review and approval by all other authors. Final translation of items of the final questionnaire into English and other languages will be performed according to EORTC guidelines [25].

#### Comparison with EORTC QLQ-C30

The issues generate in phase I were compared with the first 28 items of the EORTC QLQ-C30, omitting the two general items (“How would you rate your overall health during the past week?” and “How would you rate your overall quality of life during the past week?”). This was done in order to evaluate (1) which EORTC QLQ-C30 items are considered important for AA/PNH patients, (2) which EORTC QLQ-C30 items are considered to be unimportant, and (3) which issues are important and relevant for AA/PNH patients and are not covered by the EORTC QLQ-C30.

## Results

### Literature review

#### QoL in AA patients

While the importance of exact assessment of QoL in AA patients has been stressed by several authors [23, 27], no specific methods have been established so far and even a recently published Cochrane analysis comparing the outcome of stem cell transplantation and immunosuppressive therapy in AA patients had to admit that “Health-related quality of life questionnaires were not used in any of the included studies” they analyzed [28].

Our literature review identified a total of 17 reviews and studies performed between 1984 and 2012 in which QoL-related parameters in AA patients were reported (Table 1). In 13 of them, only surrogate parameters such as hemoglobin levels, the ability to return to work, or general functional scores such as the Karnofsky performance status scale were used [19–23, 29–36]. Only 4 studies assessed QoL by using questionnaires such as the EORTC QLQ-C30 or the SF36, and one study [24] compared differences in QoL between the two main treatment modalities, immunosuppression, and stem cell transplantation, using the quality-adjusted time without symptoms and toxicity (Q-TWiST) methodology.

**Table 1:** Literature search: Studies reporting about quality of life in adult patients with aplastic anemia

Author	Year	Number of patients evaluated; form of treatment (SCT/IST)	Country of origin	QoL instrument used
Scheinberg [21]	2011	*N* = 120; IST	USA	Surrogate parameters (blood counts, survival)
Sanders [42, 43]	2011	*N* = 49 (adult survivors of pediatric bone marrow transplantation); SCT	USA	SF36; SCL90-R; FRI; SSQSR; NBRS; MMQ
Gupta [36]	2010	*N* = 1307; SCT	Multinational	Surrogate parameters (blood counts, GvHD, survival)
Resncik [35]	2006	*N* = 13; SCT	Israel	Surrogate parameters (OS, GvHD, Karnofsky score)
Viollier [24]	2005	*N* = 207; SCT and IST	Switzerland	Q-TWiST
Frickhofen [20, 44]	2002 (1991)	*N* = 84	Germany	Surrogate parameters (blood counts, evolution of clonal/malignant disease, CSA side effects)
Matsuda [34]	2002	*N* = 10; SCT	Japan	Surrogate parameters (hemoglobin)
Goerner [33]	2002	*N* = 405; SCT	USA	Surrogate parameters (GvHD, Infections, Karnofsky score and others)
Marsh [32]	1999	*N* = 115; IST	Multinational	Surrogate parameters (blood counts, time being free of transfusions, OS)
Deeg [45]	1998	*N* = 212; SCT	USA	EORTC QLQ-C30 (plus self-developed BMT module); DBMT; POMS; WHPQ
Passweg [19]	1997	*N* = 1305; SCT	Multinational	Surrogate parameters (blood counts, GvHD, survival)
Andrykowski [46]	1995	*N* = 1 (28)**; SCT (** study comprised of 28 BMT patients including one with aplastic anemia)	USA	POMS, FLIC, PAIS, SIP
Novitzky [31]	1992	*N* = 26; IST	South Africa	Surrogate parameters (return to work, blood counts)
Najean [23]	1990	*N* = 156; failed SCT, IST (+ androgens)	France	Surrogate parameters (blood counts, occupation, marriage, pregnancies)
De Planque [22]	1989	*N* = 468; IST	Multinational	Surrogate parameters (blood counts, survival, clonal evolution)
Hinterberger [30]	1987	*N* = 23; SCT	Austria	Surrogate parameters (blood counts, GvHD)
Bayever [29]	1984	*N* = 57; SCT, IST (pediatric patients up to the age of 25)	USA	Surrogate parameters (Karnofsky score, GvHD)

*SCT* stem cell transplantation, *IST* immunosuppressive therapy, *BMT* bone marrow transplantation, *SF36* Medical Outcome Study Short Form 36 Health Survey, *SCL90-R* Symptoms Checklist Revised, *FRI* Family Relations Index, *SSQSR* Social Support Questionnaire Short Form, *NBRS* Neurobehavioral Rating Scale, *MMQ* Modified Memory Questionnaire, *OS* overall surival, *GvHD* graft-versus-host disease, *Q-TWiST* quality-adjusted time without symptoms and toxicity, *CSA* cyclosporine A, *DBMT* Demands of BMT Recovery Inventory, *POMS* Profile of Mood States, *WHPQ* Ware Health Perceptions Questionnaire, *FLIC* Functional Living Index—Cancer, *PAIS* Psychological Adjustment to Illness Scale, *SIP* Sickness Impact Profile

#### QoL in PNH patients

Six publications/studies reporting QoL and/or treatment outcomes in PNH patients could be identified (Table 2). The most reliable data on QoL stem from the pivotal eculizumab trials called TRIUMPH and SHEPHERD [11, 12] in which the EORTC QLQ-C30 questionnaire was used. Both trials also included the FACIT-Fatigue instrument after it was realized in the first 11 patients treated with eculizumab [37] in which only the C30 questionnaire had been used to assess QoL that fatigue was a major factor as well and should be measured in more detail. Two publications reported the outcome of PNH patients after allogeneic stem cell transplantation, and in these only surrogate parameters such as the occurrence of graft-versus-host disease, the normalization of blood counts, and survival data were discussed with regard to QoL aspects [38, 39].

**Table 2:** Literature search: Studies reporting about quality of life in adult patients with paroxysmal nocturnal hemoglobinuria (PNH)

Author	Year	Number of patients evaluated; form of treatment (SCT/eculizumab/other)	Country of origin	QoL instrument used
Matos-Fernandez [39]	2009	*N* = 117; SCT (review of SCT in PNH)	Multinational	Surrogate parameters (GvHD, survival)
Brodsky [12]	2008	*N* = 93; eculizumab (vs placebo)	Multinational	EORTC QLQ-C30, FACIT
Meyers [14]	2007	*N* = 29; other	Multinational	EORTC QLQ-C30, FACIT plus self-reported symptoms
Hillmen [11]	2006	*N* = 87; eculizumab (vs placebo)	Multinational	EORTC QLQ-C30, FACIT
Hill [37]	2005	*N* = 11	UK	EORTC QLQ-C30
Raiola [38]	2000	*N* = 7; SCT	Italia	Surrogate parameters (Blood counts, GvHD)

*FACIT* Functional Assessment of Chronic Illness Therapy Fatigue Scale, *GvHD* graft-versus-host disease

### Patient and physician interviews

#### Patient and physician samples

Individual patients in more than 25 German and Swiss cities were visited and personally interviewed.

During phase I, 19 patients (Table 3) and eight physicians specialized in treatment of AA/PNH were interviewed. Fifteen of the patients were female, and patients with either AA or PNH/AA comprised the majority of patients (*n* = 9 and *n* = 6), while 4 suffered from PNH without AA symptoms. Median age at diagnosis was 29.1 years (range 17.0–50.3 years). At the time of the interviews, the median age was 42.1 years. The median time from the occurrence of first disease-specific symptoms until final diagnosis was 14 months with a maximum duration of more than 5 years (range 0–61 months). Other sociodemographic characteristics and disease-specific features are given in Table 3.

**Table 3:** Patient characteristics of the patients interviewed during phases I and II

	Phase I (*n* = 19)	Phase II (*n* = 30)
Sex		
Female	15 (79%)	15 (50%)
Male	4 (21%)	15 (50%)
Disease^a^		
PNH	4 (21%)	10 (33%)
AA	9 (47%)	10 (33%)
AA/PNH	6 (32%)	10 (33%)
Previous therapies^a^		
BMT	3 (16%)	2 (7%)
CSA	13 (68%)	20 (67%)
ATG	13 (68%)	17 (57%)
eculizumab	6 (32%)	15 (53%)
Age at diagnosis (in years)^a^		
Median; mean; range	29.1; 30.7; 17.0–50.3	31.3; 43.2; 13.9–60.3
Age at the time of interview (in years)		
Median; mean; range	42.1; 40.7; 25.1–61.3	43.7; 43.3; 18.9–73.5
Time from first symptoms until first physician contact (in months)^a^		
Median; mean; range	0; 9; 0–56	0; 3; 0–24
Time from first physician contact to final diagnosis (in months)^a^		
Median; mean; range	5; 14; 0–56	2; 6; 0–37

*CSA* Cyclosporin A, *BMT* bone marrow transplantation, *ATG* Antithymocyte Globulin

^a^Self-reported

All of the eight physicians were hematologists by training, seven worked in a clinic; four of them were consultants, three were heads of hematology or stem cell transplantation departments, and one was a registered hematologist working in a private office. Six physicians were from Germany, one from Switzerland, and one from Austria.

Based on the experiences from phase I, special efforts were made to include patients with a more heterogeneous distribution regarding sex, disease, and time since diagnosis in phase II. Thirty patients were contacted and agreed to participate, 10 of each disease entity, and half of them being male. Their median age at diagnosis was 31.3 years and their median age at the time of the interview was 43.7 years. The median time from first disease-specific symptom to final diagnosis was 4 months (range 0–61 months).

Fourteen physicians participated in phase II (of whom five also took part in phase I), all of them working in a clinic at a university hospital (four as head of the respective department, ten as consultants), 13 from Germany, and one from Austria.

#### QoL issues

Out of the interviews in phase I, 649 QoL issues were derived. Similar issues were combined into one, so that the list could be condensed to 175 separate issues. The majority (141/175) concerned general health-related QoL problems faced by patients with AA and/or PNH, while the remaining 34 issues applied to problems experienced with healthcare (HC). It was therefore decided to develop two questionnaires, one for QoL and one for HC.

#### Relevance of issues

Of the 175 issues derived from phase I, 97 issues were rated important (see Supplementary Table 5) according to at least two relevance criteria (see rating of issues within the “Methods” section). Of these 97 issues, 77 concerned QoL and 20 healthcare problems.

Fifteen issues met all four relevance criteria, 31 issues met three criteria, and the remaining 51 issues met two criteria. Neither patients nor physicians mentioned any missing issue.

#### Differences between AA and PNH patients

In general, issues were rated comparable between patients with AA compared to patients with PNH. The only QoL issue rated >2 points higher by patients with AA compared to patients with PNH was “fear of therapy failure.” In contrast, PNH-only patients rated the QoL issues “appearance” and “sleep disorder/disturbance” >2 points higher.

In regards to healthcare issues, PNH patients rated the issues “it helps having a personal bond with my primary doctor” (individual support), “it is important having doctors agreeing on treatment” (physician network), “getting a second opinion”, and “problems within the social legislation” >2 points higher than patients with AA when looking at the median.

#### Comparison with the EORTC QLQ-C30 issues

Comparison of the EORTC QLQ-C30 items with the 175 issues generated in phase I revealed that only 16 of them were brought up or rated as important by AA and/or PNH patients. Five EORTC QLQ-C30 items were not mentioned at all—these being items rather typical for cancer patients or often experienced during chemotherapy such as lack of appetite, constipation or difficulties remembering things—while seven others were mentioned during the interviews in phase I but rated as unimportant in phase II (Table 4).

**Table 4:** Comparison of issues from phase I with items from the EORTC QLQ-C30 questionnaire

EORTC QLQ-C30 items	Patient rating (mean; median)
Mentioned and rated important	
1. Do you have any trouble doing strenuous activities, like carrying a heavy shopping bag or a suitcase?	3.2; 3.0
2. Do you have any trouble taking a long walk?	2.6; 3.0
3. Do you need to stay in bed or a chair during the day?	2.3; 2.0
4. Were you limited in doing either your work or other daily activities?	2.4; 2.0
5. Were you limited in pursuing your hobbies or other leisure time activities?	2.4; 2.0
6. Were you short of breath?	2.9; 3.0
7. Did you need to rest?	3.3; 4.0
8. Have you had pain?	2.5; 3.0
9. Did pain interfere with your daily activities?	2.3; 3.0
10. Have you had trouble sleeping?	2.2; 2.0
11. Have you felt weak?	2.6; 3.0
12. Were you tired?	3.1; 3.0
13. Have you had difficulty in concentrating on things, like reading a newspaper or watching television?	2.4; 2.0
14. Did you feel tense?	2.3; 2.0
15. Did you worry?	2.7; 3.0
16. Did you feel depressed?	2.4; 2.0
Mentioned and rated unimportant	
1. Do you have any trouble taking a short walk outside of the house?	1.8; 1.0
2. Do you need help with eating, dressing, washing yourself or using the toilet?	1.2; 1.0
3. Have you felt nauseated?	1.6; 1.0
4. Have you had diarrhea?	1.5; 1.0
5. Has your physical condition or medical treatment caused you financial difficulties?	1.9; 1.0
6. Has your physical condition or medical treatment interfered with your social activities?	1.7; 1.0
7. Has your physical condition or medical treatment interfered with your family life?	1.7; 1.0
Not mentioned	
1. Have you lacked appetite?	n.a.
2. Have you vomited?	n.a.
3. Have you been constipated?	n.a.
4. Did you feel tense?	n.a.
5. Have you had difficulty remembering things?	n.a.

*n.a.* not applicable

#### Generation of items

Modifications in phrasing finally led to two questionnaires: one with 77 items regarding QoL and one with 20 items regarding healthcare.

## Discussion

Here, we report the first two phases of the generation of a specific QLQ-AA/PNH assessment tool, which resulted in the development of a preliminary questionnaire. Looking at the literature search results, it became obvious that no issues could be derived from previous studies in AA patients. Even the recent large seminal trial comparing horse and rabbit ATG for treatment of severe and very severe aplastic anemia including 120 patients did not cover quality of life [21].

The situation for patients with PNH is somewhat better in that both the EORTC QLQ-C30 and the FACIT-Fatigue instruments have been used to evaluate quality of life in patients treated with the complement inhibitor eculizumab [11, 12] and are now used routinely for patients enrolled into the International PNH registry [15]. This enabled researchers to come up with the first real-life data about patients with PNH demonstrating that these patients indeed experience a lower global health status compared to the general population [15]. This publication also showed that suffering from PNH leads to problems at patients’ workplace and repeated hospitalizations in a proportion of patients mirroring the chronicity of the disease under targeted treatment.

So far, only one survey evaluated the applicability of the QLQ-C30 (and the FACIT-Fatigue) instrument in patients with PNH [14] and [40]. In this study, the 29 patients from Spain, France, the UK, and the USA agreed that the FACIT-Fatigue instrument was highly relevant and adequate in assessing the level of fatigue of which all but one of the patients complained; however, the QLQ-C30 was rated as being not as relevant in assessing other quality of life aspects. While the authors stated that their “study confirms the validity of the FACIT-Fatigue and the EORTC QLQ-C30 questionnaires in this patient population and their routine use should be considered in the management of patients with PNH,” the results of our interviews and the comparison of the derived issues with the items of the QLQ-30 underline that QoL aspects of patients with PNH and/or AA seem to be inappropriately captured with available EORTC-QoL tools. Even the EORTC Quality of life group (QLG) states that “while the EORTC QLQ-C30 is an important tool for assessing the generic aspects of QoL, it has limitations” and “that it should be supplemented by additional modules” (S126–S128 in [13]). This has been successfully carried out for 17 malignancies including colorectal carcinoma, multiple myeloma, breast cancer and others. However, all these disease entities are cancers, and their incidence is markedly higher than AA and/or PNH.

We found several new QoL aspects such as “constant fear of infection” and fear of “variations of blood counts,” “dependency on time-consuming therapies,” and “emotional strain through endlessness of disease” that are relevant for a majority of AA/PNH patients. It therefore proved useful to continue with the development of an AA/PNH-specific QoL questionnaire. We used the well-established EORTC questionnaire development guidelines [25] for creating a specific and comprehensive QoL questionnaire for patients with two non-malignant hematologic diseases, though non-malignant is a somewhat misleading term for AA and PNH as it is known that the mortality and morbidity of these bone marrow failure syndromes is comparable to those of malignant hematologic diseases such as chronic myeloid leukemia (CML), multiple myeloma (MM), or some forms of non-Hodgkins lymphoma (NHL), especially in the era of targeted and individualized therapies [40].

The fact that twelve of the QLQ-C30 items were not rated relevant or not even mentioned at all despite the fact that during phases I and II 49 AA/PNH patients and 17 physicians with long-time experience in medical care of these patients were thoroughly questioned shows that there clearly is a difference between cancer patients and our patient group. In addition, while it was planned to develop a QoL questionnaire with a maximum of 30 to 35 items, patients and physicians rated 77 issues concerning health-related quality of life as being so important that they should be included into the pre-final instrument.

Only a few issues were rated differently between patients with AA and PNH. The “fear of therapy failure” as being the one issue rated high by AA patients, while almost being ignored by PNH patients reflects the different treatment modalities with IST being applied to AA patients unfortunately still has a failure rate of almost 30% with a cumulative incidence of relapse in up to 28% of patients despite the high rate of primary responders of almost 70% at 6 months in patients treated with horse ATG and CSA [21].

The issues being rated significantly higher in patients with PNH mostly reflect the problems faced by a patient group with a chronic disease under a treatment that effectively tackles the disease without causing a real cure as is the fact with the complement inhibitor eculizumab. Eculizumab effectively relieves symptoms and prevents morbidity and mortality through prevention of hemolysis and thrombosis; however, it does so by inhibiting complements effector phase but does not alter the primary GPI deficiency [41]. Thus, patients have to receive regular eculizumab infusions every 2 weeks and while often experience an amelioration of their symptoms and general performance status, they still have to face psychosocial strain in communication with their friends and relatives and even more so with the legislative social system when it comes to provisioning of unemployment and healthcare benefits. This is also reflected in the fact that the rating of issues by patients and physicians directed us to come up with 20 issues, which mainly covered healthcare issues and did not concern physical quality of life issues. It is noteworthy that these extra 20 issues came up while we were asking explicitly for QoL concerns of the patients. Our assumption is that this is due to the rarity of the disease, which makes it especially difficult for the patients to find adequate treatment and care.

The differences between AA and PNH patients, however, were not that substantial that it would be advisable to create two different questionnaires for the two disease entities.

Taken together, the first and second phase of this study clearly demonstrated the need for an AA/PNH-disease-specific QoL tool and the feasibility of developing such a tool by using slightly modified EORTC guideline development specifications. The support of patient advocacy groups in such project with ultra-rare diseases proved especially helpful.

In phase III, the questionnaire will now be pilot-tested. Eventually, the QLQ-AA/PNH questionnaire will be validated and psychometrically tested (phase IV).

## Electronic supplementary materials

Below is the link to the electronic supplementary material.

ESM 1 Supplemental Tables 5.1 and 5.2. The following table shows the 141 Issues concerning the patients’ quality of life except for the issues concerning healthcare problems. Shown are the number of fulfilled relevance criteria, the mean and median ranking of importance for all patients (*n* = 30), and the median ranking for patients with PNH (*n* = 10), for patients with AA (*N* = 10) and for patients with AA/PNH (*n* = 10). Issues, rated ≥2 points higher (median) from AA patients compared to PNH patients are highlighted in red (I10); issues, rated ≥2 points higher (median) from PNH patients compared to AA patients are highlighted in blue (I67, I70). (DOC 706 kb)
