# Supplementary material for: Development of a disease-specific quality of life questionnaire for patients with aplastic anemia and/or paroxysmal nocturnal hemoglobinuria (QLQ-AA/PNH)—report on phases I and II
Source: Ann Hematol. 2016 Nov 11;96(2):171–81. doi: 10.1007/s00277-016-2867-8 (PMC5226974; doi:10.1007/s00277-016-2867-8)
Supplement: Supplementary file 1 — Supplemental Tables 5.1 and 5.2. The following table shows the 141 Issues concerning the patients’ quality of life except for the issues concerning healthcare problems. Shown are the number of fulfilled relevance criteria, the mean and median ranking of importance for all patients (n = 30), and the median ranking for patients with PNH (n = 10), for patients with AA (N = 10) and for patients with AA/PNH (n = 10). Issues, rated ≥2 points higher (median) from AA patients compared to PNH patients are highlighted in red (I10); issues, rated ≥2 points higher (median) from PNH patients compared to AA patients are highlighted in blue (I67, I70). (DOC 706 kb) [file 277_2016_2867_MOESM1_ESM.doc]

**Development of a Disease Specific Quality of Life Questionnaire for Patients with Aplastic Anemia and/or Paroxysmal Nocturnal Hemoglobinuria (QLQ-AA/PNH) – report on Phase I & II**

Martha Groth^1^, Susanne Singer^2^, Cathrin Niedeggen^1^, Andrea Petermann-Meyer^1^, Alexander Röth^3^, Hubert Schrezenmeier^4^, Britta Höchsmann^4^, Tim H. Brümmendorf^1^, Jens Panse^1^

^1^Department of Oncology, Hematology, Hemostaseology and Stem Cell Transplantation, Medical Faculty, University Hospital RWTH Aachen, Aachen, Germany

^2^Division of Epidemiology and Health Services Research, Institute of Medical Biostatistics, Epidemiology and Informatics, University Medical Centre Mainz, Germany

^3^Department of Hematology, University Hospital Essen, University of Duisburg-Essen, Essen, Germany

^4^Institute of Clinical Transfusion Medicine and Immunogenetics, German Red Cross Blood Transfusion Service and Institute of Transfusion Medicine, University of Ulm, Ulm, Germany

**Corresponding author:**

Jens Panse, MD

Department of Oncology, Hematology, Hemostaseology and Stem Cell Transplantation,

Medical Faculty, University Hospital RWTH Aachen

Pauwelsstr. 30, 52074 Aachen,

Phone: +49-241-89947; Fax: +49-241-82449; E-mail: jpanse@ukaachen.de

**Keywords:**  aplastic anemia, paroxysmal nocturnal hemoglobinuria, quality of life, bone marrow failure syndromes

**Acknowledgments:**

The project was supported by a grant from the registered association Aplastische Anämie e.V. (www.aplastische-anämie.de), which is a patient-advocacy-group and part of the German Leukemia- and Lymphoma Support Group (DLH). We thank especially Michael Kaiser and Ulrike Scharbau of the Aplastische Anämie e.V..

We are grateful to all the patients and physicians who took part in our study for their generous time and willingness to participate.

**Abstract:**

**INTRODUCTION:** Acquired aplastic anemia (AA) and paroxysmal nocturnal hemoglobinuria (PNH) are interrelated ultra-rare diseases. Quality of life (QoL) evaluation tools used in studies for AA and PNH are unspecific and designed for cancer patients (e.g., the European Organization for Research and Treatment of Cancer Quality of Life Questionnaire, EORTC QLQ-C30). Given the complexity of AA and PNH, variation in symptoms and treatments, younger age of many pts, and the fact that AA and PNH are not classified as malignant diseases, it is likely that cancer-specific questionnaires are inappropriate. **METHODS:** Generation of an AA/PNH specific QoL questionnaire (QLQ-AA/PNH), performed according to EORTC guidelines. QoL issues were obtained from the literature and interviews with pts and physicians (phase I), then ranked by patients and physicians. In phase II items were created. **RESULTS:** Patients in more than 25 German and Swiss cities were interviewed face to face. In phase I, interviews of 19 pts and 8 physicians specialized in AA/PNH-treatment resulted in 649 QoL issues; these were condensed to 175, and graded according to their importance by 30 pts and 14 physicians (phase II). 5 physicians took part in phase I and II. Altogether, 97 issues were rated important. 12 EORTC QLQ-C30 items were not rated important, while several new QoL aspects were brought up. Modifications in wording and phrasing led to two questionnaires with 77 items regarding general QoL-aspects and 20 items regarding medical care. **CONCLUSION:** Important QoL aspects of PNH/AA pts are inappropriately captured with available QoL tools. Developing a new QoL questionnaire specific for this patient group is warranted.

**INTRODUCTION:**

Both, acquired Aplastic Anemia (AA) and Paroxysmal Nocturnal Hemoglobinuria (PNH) are ultra-rare diseases with a yearly incidence within the western hemisphere of 1.3 to 2 per million . The number of patients newly developing AA, PNH or overlap syndromes e.g. within Germany is estimated to be 250 per year, however, not all of them are properly diagnosed so that the actual number of newly diagnosed patients is somewhat lower. Age distribution for AA shows a bimodal distribution with a peak in young adults, while the mean age at diagnosis of PNH is between 30 and 45 years. PNH and AA are interrelated diseases and cannot be viewed separately as both belong to the group of bone marrow failure syndromes (BMFS) .

For patients with PNH, the median overall survival before the introduction of the complement inhibitor Eculizumab was between 10 and 22 years with about a third of patients dying within the first ten years after diagnosis whereas now basically all patients survive the first year and data from the long-term safety and efficacy of Eculizumab in 195 patients with hemolytic PNH show that consequent complement inhibitor treatment results in a 3-year survival estimate of 97.6%, i.a. as in age-matched controls . Allogeneic bone marrow transplantation (BMT), rarely used in patients with PNH, is the treatment of choice for patients below the age of 40 with severe AA and an available HLA-identical sibling . Treatment decisions in AA for patients without an HLA-identical sibling, older patients or patients not responding to first-line immunosuppressive therapy (IST) are more complex and involve further courses of IST, BMT from alternative donor sources or experimental treatment approaches .

While considerable research has been performed and published with regard to the patho-physiology and treatment of AA and PNH, much less is known regarding both diseases’ psycho-social issues and their impact on quality of life (QoL).

So far the only reports on QoL for PNH stem from the original Eculizumab trials (TRIUMPH and SHEPHERD) using the European Organization for Research and Treatment of Cancer Quality of Life Questionnaire (EORTC QLQ)-C30 including 87 patients in the TRIUMPH and 93 patients in the SHEPHERD trial. In addition to QoL, fatigue as a major clinical symptom of patients with PNH was prospectively evaluated in both trials using the Functional Assessment of Chronic Illness Therapy Fatigue Instrument (FACIT-Fatigue). Both trials demonstrated that, compared to placebo, treatment with Eculizumab resulted in clinically significant improvements in regards to QoL and fatigue.

In another small series with 29 patients, fatigue and abdominal pain were reported as relevant QoL issues .

Both questionnaires, the EORTC QLQ-C30 and the FACIT-Fatigue are now routinely used within the PNH-registry, a prospectively documenting international registry that has been established following a post-marketing commitment of the Eculizumab manufacturer Alexion Pharmaceuticals Inc., requested by both the EMA (European Medicines Agency) and the FDA (Food and Drug Administration) .

In AA, QoL assessment is even less standardized and research so far mainly investigated sequelae from bone marrow transplantation . Even large studies on treatment outcome mainly used surrogate parameters such as hemoglobin levels and other blood counts or survival to investigate patient burden, but QoL was not measured with validated self-assessed instruments by patients . Few studies evaluated QoL aspects in non-transplanted AA patients , and there is only one report comparing QoL aspects of patients after BMT to those receiving immunosuppressive therapy retrospectively . However, the QoL-evaluation tools used in all these studies were again either surrogate parameters such as treatment toxicity, transfusions and drug requirements, hematologic counts or rates of employment after long-time follow-up or were unspecific for AA-patients and transferred from evaluations in cancer patients e.g. by using the quality-adjusted time without symptoms and toxicity (Q-TWiST) methodology.

Given the complexity of AA and PNH, the variation in symptoms and different treatment approaches (IST, BMT, complement inhibition with Eculizumab and others and eventually sequences of all in some pts.), the young age of the patients, and the fact that marrow failure syndromes are not classified as malignant diseases, it is likely that the cancer-specific tools used so far are inappropriate to adequately assess the QoL and illness intrusiveness in this group of patients.

Only few experts in the field of PNH can be found and even within the hematologic community, PNH and AA might be underestimated in regards to morbidity and mortality, leading to inadequate or delay of treatment.

We hypothesize that lack of adequate information and counseling, lack of support systems and the fragmentation of services as well as the varying quality of medical services lead to a profound impact on psycho-social well-being of patients with AA/PNH. Physicians probably often underestimate the resulting disease burden and impacts on QoL.

Based on these considerations, we therefore initiated the development of a AA/PNH-specific QoL-instrument (QLQ-AA/PNH) according to EORTC-guidelines . The objectives of this study were to identify symptoms and QoL issues reported by patients with AA and/or PNH and identified by physicians working as specialists in the field of AA/PNH treatment and to determine how both groups rate their importance. According to the EORTC quality of life group (EORTC QLG), an issue is a term used to describe and identify a QoL domain that is potentially affected by a disease (malignancy) and/or its treatment .

**METHODS:**

**Phase I (Generation of issues)**

***Literature review:***

From March 2012 to Mai 2012, a systematic literature search was performed using the Medline database and the Website of the American Society of Hematology (ASH) to search the abstracts of the annual ASH meeting. Search criteria included the following terms: aplastic anemia, aplastic syndrome, paroxysmal nocturnal hemoglobinuria plus one of the following search terms: quality of life, therapy, bone marrow transplantations, immunosuppression, Antithymocyte Globulin (ATG), Eculizumab, patient perspective, symptoms.

Medline search was restricted to articles published between 1985 and 2012 and abstracts from the annual ASH meeting were restricted to be published between 2004 and 2012.

All publications and abstracts were carefully searched for QoL issues mentioned and QoL instruments used.

***Interviews of patients and physicians specialized in treatment of PNH/AA:***

Because of the rarity of the disease, special efforts were made to include patients with different disease stages, educational background and geographical region.

Patients for phase I interviews were approached mainly via physicians and via patient-advocacy groups. The internet-forum [www.pnh-aa.info](http://www.pnh-aa.info/), founded as a self-help initiative, provides a bulletin board for registered members with links to reference centers and specialists and downloadable information brochures as well as the registered association Aplastische Anämie e.V. (www.aplastische-anämie.de), which is a patient-advocacy-group and part of the German Leukemia- and Lymphoma Support Group (DLH). Both self-help-groups announced our research projects on their website and encouraged patients to take part.

Physicians known as specialists for treatment of AA/PNH, e.g. actively involved in the development of treatment guidelines within Germany, Austria and Switzerland, were contacted by two of the authors (T.H.B and J.P) and patients treated within centers were approached by these physicians.

Additional patients were enrolled during two patient conferences, through a facebook website of one of the patients and by word-of-mouth from other patients.

Ethical approval was obtained from the Ethical committee of the Medical Faculty RWTH Aachen University and an informed consent letter, which was signed by each participating patient, was phrased in consultation with the data safety manager of the University Hospital RWTH Aachen.

After patients sent back their signed informed consent, one of the authors (M.G.) contacted them and interviews were appointed. Most patients wanted to be interviewed at home alone, however two interviews were done in pairs and one group of patients (n=4) was interviewed together in the form of a focus group.

The interviews were open and qualitative. At the beginning of each interview, patients were asked to give a brief overview of their disease history including first symptoms, time to first physician contact, time to diagnosis and treatment history. Patients were sub-grouped into those with predominant AA-features, predominant PNH-features and those with overlapping PNH/AA features. In addition, patients were asked to give details regarding their supportive care needs, (e. g. information, support by medical staff, psychosocial counseling, patient support groups etc.) and potential problems experienced due to the non-specificity of symptoms and the local health care provision (e. g. delay in diagnosis, lack of local availability of disease specific experts, appreciation of QoL problems by health care professionals).

Patients were invited to report about the diseases’ impact on their quality of life without restricting themselves. All interviews were audio-recorded and subsequently transcribed verbatim. During the focus group interview, issues were recorded on a flip chart by the investigator (M.G.) and discussed within the group. The session was videotaped and subsequently transcribed in order not to miss any potential issue that was raised. Two reviewers then independently analyzed the transcripts to identify OoL issues mentioned by the patients. Identified issues were then compared and kept if both reviewers identified them as potentially affecting QoL.

Physician interviews were performed accordingly. With the exception of one interview, which was done by telephone, all were done in person by one author (M.G.). Issues raised by the physicians were then compared to the already collected issues derived from patient interviews and new issues were added to the compilation.

Issues were sorted according to their content into separate categories: physical, psychological, social, financial, health care/therapy related. Three reviewers (M.G., S.S., J.P.) separately evaluated each issue and rated them as follows: "keep" (keep issue as is), "change" (keep issue, but rephrase) "delete" (delete issue). Issues rated "keep" or "change" from ≥ 2 reviewers were kept, whereas issues rated "delete" from all three reviewers were deleted without further discussion. Issues rated "keep" or "change" in addition two times "delete" were kept if the reviewer who had performed the interviews rated them relevant after discussion with the other two reviewers.

**Phase II (rating of issues)**

***Review and rating of issues***

From the interviews, a list of QoL issues was generated according to . Within a semi-structured interview, patients - different from those in phase I - and physicians – five also being interviewed in phase I - were asked to prioritize the 25 most salient issues and rate all issues on a Likert-scale (very important [4 points], important [3 points], moderately important [2 points], unimportant [1 point]).

Issues were considered relevant with a high priority for inclusion if ≥ 2 of the following four criteria were fulfilled: The mean rating of an issue by the total patient sample was ≥ 2.0; the mean rating of a patient subgroup (AA, PNH/AA, or PNH) was ≥ 2.5; ≥ 20% of patients ranked an issue among the top 25 issues; ≥ 30% of physicians ranked an issue among the top 25 issues. Patients and physicians were also asked if any issues were missing. In contrast to EORTC-guidelines, patients and physicians were asked to rate issues as being important or not considering the whole course of their disease and not just the last couple of weeks. This was done because PNH and AA patients often experience their diseases as a chronic, long lasting state with various “disease flares” and patients after bone marrow transplantation might experience completely different disease features and treatment related morbidities before versus after the transplantation procedure.

***Generation of items***

All issues fulfilling the above mentioned criteria were reworded into items so that they can be completed with the same response categories as it is required for a self-administered questionnaire. The wording was suggested by one author (M.G.) and reviewed by two authors (S.S., J.P.) until a consensus was reached.

All interviews, recordings, transcriptions, and generation of issues and items were done in German. For the purpose of publication this manuscript translation of issues and items into English language was done by one author (J.P.) with review and approval by all other authors. Final translation of items of the final questionnaire into English and other languages will be performed according to EORTC guidelines .

***Comparison with EORTC QLQ-C30***

The issues generate in phase I were compared with the first 28 items of the EORTC QLQ-C30, omitting the two general items (“How would you rate your overall health during the past week?” and “How would you rate your overall quality of life during the past week?”). This was done in order to evaluate 1. which EORTC QLQ-C30 items are considered important for AA/PNH-patients, 2. which EORTC QLQ-C30 items are considered to be unimportant, and 3. which issues are important and relevant for AA/PNH-patients and are not covered by the EORTC QLQ-C30.

**RESULTS:**

**Literature Review:**

***QoL in AA patients:***

While the importance of exact assessment of QoL in AA patients has been stressed by several authors , no specific methods have been established so far and even a recently published Cochrane analysis comparing the outcome of stem cell transplantation and immunosuppressive therapy in AA patients had to admit that “Health-related quality of life questionnaires were not used in any of the included studies” they analyzed .

Our literature review identified a total of 17 reviews and studies performed between 1984 and 2012 in which QoL-related parameters in AA patients were reported (Table 1). In 13 of them, only surrogate parameters such as hemoglobin levels, the ability to return to work or general functional scores such as the Karnofsky performance status scale where used . Only 4 studies assessed QoL by using questionnaires such as the EORTC QLQ-C30 or the SF36, and one study compared differences in QoL between the two main treatment modalities, immunosuppression and stem cell transplantation, using the quality-adjusted time without symptoms and toxicity (Q-TWiST) methodology.

***QoL in PNH patients***

Six publications/studies reporting QoL and/or treatment outcomes in PNH patients could be identified (Table 2). The most reliable data on QoL stem from the pivotal Eculizumab trials called TRIUMPH and SHEPHERD in which the EORTC QLQ-C30 questionnaire was used. Both trials also included the FACIT-Fatigue instrument after it was realized in the first 11 patients treated with Eculizumab in which only the C30 questionnaire had been used to asses QoL that fatigue was a major factor as well and should be measured in more detail. Two publications reported outcome of PNH patients after allogeneic stem cell transplantation and in these only surrogate parameters such as the occurrence of Graft-versus-Host-Disease, the normalization of blood counts and survival data were discussed with regard to QoL aspects .

**Patient and Physician interviews:**

***Patient and physician samples***

Individual patients in more than 25 German and Swiss cities were visited and personally interviewed.

During phase I, 19 patients (Table 3) and eight physicians specialized in treatment of AA/PNH were interviewed. 15 of the patients were female, and patients with either AA or PNH/AA comprised the majority of patients (n=9 and n=6), while 4 suffered from PNH without AA symptoms. Median age at diagnosis was 29.1 years (range 17.0 – 50.3 years). At the time of the interviews, the median age was 42.1 years. The median time from the occurrence of first disease specific symptoms until final diagnosis was 14 months with a maximum duration of more than five years (range 0 – 61 months). Other socio-demographic characteristics and disease specific features are given in Table 3.

All of the eight physicians were hematologists by training, seven worked in a clinic; four of them were consultants, three were heads of hematology or stem cell transplantation departments and one was a registered hematologist working in a private office. Six physicians were from Germany, one from Switzerland and one from Austria.

Based on the experiences from phase I, special efforts were made to include patients with a more heterogeneous distribution regarding sex, disease, and time since diagnosis in phase II. Thirty patients were contacted and agreed to participate, 10 of each disease entity, and half of them being male. Their median age at diagnosis was 31.3 years and their median age at the time of the interview 43.7 years. The median time from first disease specific symptom to final diagnosis was four months (range 0 – 61 months).

14 physicians participated in phase II (of whom five also took part in phase I), all of them working in a clinic at a university hospital (four as head of the respective department, ten as consultants), thirteen from Germany and one from Austria.

***QoL issues***

Out of the interviews in phase I, 649 QoL issues were derived. Similar issues were combined into one, so that the list could be condensed to 175 separate issues. The majority (141/175) concerned general health related QoL-problems faced by patients with AA and/or PNH, while the remaining 34 issues applied to problems experienced with health care (HC). It was therefore decided to develop two questionnaires, one for QoL and one for HC.

***Relevance of issues***

Of the 175 issues derived from phase I, 97 issues were rated important (see suppl. Table 5) according to at least two relevance criteria (see rating of issues within the Methods section). Of these 97 issues, 77 concerned QoL and 20 health care problems.

15 issues met all four relevance criteria, 31 issues met three criteria and the remaining 51 issues met two criteria. Neither patients nor physicians mentioned any missing issue.

***Differences between AA and PNH patients***

In general, issues were rated comparable between patients with AA compared to patients with PNH. The only QoL issue rated >2 points higher by patients with AA compared to patients with PNH was “fear of therapy failure”. In contrast, PNH-only patients rated the QoL issues “appearance” and “sleep disorder/disturbance” > 2 points higher.

In regards to health care issues, PNH patients rated the issues “it helps having a personal bond with my primary doctor” (individual support), “it is important having doctors agreeing on treatment” (physician network), “getting a second opinion”, and “problems within the social legislation” > 2 points higher than patients with AA when looking at the median.

***Comparison with the EORTC QLQ-C30 issues***

Comparison of the EORTC QLQ-C30 items with the 175 issues generated in phase I revealed that only 16 of them were brought up or rated as important by AA and/or PNH patients. Five EORTC QLQ-C30 items were not mentioned at all – these being items rather typical for cancer patients or often experienced during chemotherapy such as lack of appetite, constipation or difficulties remembering things - while seven others were mentioned during the interviews in phase I but rated as unimportant in phase II (Table 4).

***Generation of items***

Modifications in phrasing finally led to two questionnaires: one with 77 items regarding QoL and one with 20 items regarding health care.

**DISCUSSION:**

Here we report the first two phases of the generation of a specific QLQ-AA/PNH assessment tool, which resulted in the development of a preliminary questionnaire. Looking at the literature search results it became obvious that no issues could be derived from previous studies in AA patients. Even the recent large seminal trial comparing horse and rabbit ATG for treatment of severe and very severe aplastic anemia including 120 patients did not cover quality of life .

The situation for patients with PNH is somewhat better in that both the EORTC QLQ-C30 and the FACIT-Fatigue instruments have been used to evaluate quality of life in patients treated with the complement inhibitor Eculizmab and are now used routinely for patients enrolled into the International PNH-registry . This enabled researchers to come up with the first real-life data about patients with PNH demonstrating that these patients indeed experience a lower global health status compared to the general population . This publication also showed that suffering from PNH leads to problems at patients’ workplace and repeated hospitalizations in a proportion of patients mirroring the chronicity of the disease under targeted treatment.

So far, only one survey evaluated the applicability of the QLQ-C30 (and the FACIT-Fatigue) instrument in patients with PNH and [[40](#_ENREF_43)]. In this study, the 29 patients from Spain, France, the UK and the USA agreed that the FACIT-Fatigue instrument was highly relevant and adequate in assessing the level of fatigue of which all but one of the patients complained; however, the QLQ-C30 was rated as being not as relevant in assessing other quality of life aspects. While the authors stated that their “study confirms the validity of the FACIT-Fatigue and the EORTC QLQ-C30 questionnaires in this patient population and their routine use should be considered in the management of patients with PNH”, the results of our interviews and the comparison of the derived issues with the items of the QLQ-30 underline that QoL-aspects of patients with PNH and/or AA seem to be inappropriately captured with available EORTC-QoL tools. Even the EORTC Quality of life group (QLG) states that “while the EORTC QLQ-C30 is an important tool for assessing the generic aspects of QoL, it has limitations” and “that it should be supplemented by additional modules” (S126 – S128 in ). This has been successfully carried out for 17 malignancies including colorectal carcinoma, multiple myeloma, breast cancer and others. However, all these disease entities are cancers, and their incidence is markedly higher than AA and/or PNH.

We found several new QoL aspects such as “constant fear of infection” and fear of “variations of blood counts”, “dependency on time-consuming therapies”, and “emotional strain through endlessness of disease” that are relevant for a majority of AA/PNH patients. It therefore proved useful to continue with the development of an AA/PNH-specific QoL questionnaire. We used the well-established EORTC-questionnaire development guidelines for creating a a specific and comprehensive QoL-questionnaire for patients with two non-malignant hematologic diseases, though non-malignant is a somewhat misleading term for AA and PNH as it is known that the mortality and morbidity of these bone marrow failure syndromes is comparable to those of malignant hematologic diseases such as chronic myeloid leukemia (CML), Multiple Myeloma (MM) or some forms of Non-Hodgkins-Lymphoma (NHL), especially in the era of targeted and individualized therapies .

The fact that twelve of the QLQ-C30 items were not rated relevant or not even mentioned at all despite the fact that during phases I and II 49 AA/PNH patients and 17 physicians with long time experience in medical care of these patients were thoroughly questioned shows that there clearly is a difference between cancer patients and our patient group. In addition, while it was planned to develop a QoL-questionnaire with a maximum of 30 to 35 items, patients and physicians rated 77 issues concerning health related quality of life as being so important that they should be included into the pre-final instrument.

Only a few issues were rated differently between patients with AA and PNH. The “fear of therapy failure” as being the one issue rated high by AA patients, while almost being ignored by PNH patients reflects the different treatment modalities with IST being applied to AA patients unfortunately still has a failure rate of almost 30% with a cumulative incidence of relapse in up to 28% of patients despite the high rate of primary responders of almost 70% at 6 months in patients treated with horse ATG and CSA .

The issues being rated significantly higher in patients with PNH mostly reflect the problems faced by a patient group with a chronic disease under a treatment that effectively tackles the disease without causing a real cure as is the fact with the complement inhibitor Eculizumab. Eculizumab effectively relieves symptoms and prevents morbidity and mortality through prevention of hemolysis and thrombosis; however, it does so by inhibiting complements effector phase but does not alter the primary GPI-deficiency . Thus, patients have to receive regular Eculizumab infusions every two weeks and while often experience an amelioration of their symptoms and general performance status, they still have to face psycho-social strain in communication with their friends and relatives and even more so with the legislative social system when it comes to provisioning of unemployment and health care benefits. This is also reflected in the fact that the rating of issues by patients and physicians directed us to come up with 20 issues, which mainly covered health care issues and did not concern physical quality of life issues. It is noteworthy that these extra 20 issues came up while we were asking explicitly for QoL concerns of the patients. Our assumption is that this is due to the rarity of the disease, which makes it especially difficult for the patients to find adequate treatment and care.

The differences between AA and PNH patients, however, were not that substantial that it would be advisable to create two different questionnaires for the two disease entities.

Taken together, the first and second phase of this study clearly demonstrated the need for an AA/PNH-disease specific QoL-tool and the feasibility of developing such a tool by using slightly modified EORTC-guideline development specifications. The support of patient advocacy groups in such project with ultra-rare diseases proved especially helpful.

In phase III, the questionnaire will now be pilot-tested. Eventually, the QLQ-AA/PNH questionnaire will be validated and psychometrically tested (phase IV).

**Table 1** Literature search: Studies reporting about quality of life in adult patients with aplastic anemia

| **Author** | **Year** | **No. of patients evaluated; form of treatment (SCT/IST)** | **Country of origin** | **QoL instrument used** |
| --- | --- | --- | --- | --- |
| Scheinberg | 2011 | N = 120; IST | USA | Surrogate parameters (blood counts, survival) |
| Sanders | 2011 | N=49 (adult survivors of pediatric bone marrow transplantation); SCT | USA | SF36; SCL90-R; FRI; SSQSR; NBRS; MMQ |
| Gupta | 2010 | N = 1307; SCT | Multinational | Surrogate parameters (blood counts, GvHD, survival) |
| Resncik | 2006 | N=13; SCT | Israel | Surrogate parameters (OS, GvHD, Karnofsky score) |

| Viollier | 2005 | N=207; SCT and IST | Switzerland | Q-TWiST |
| --- | --- | --- | --- | --- |

| Frickhofen | 2002 (1991) | N = 84 | Germany | Surrogate parameters (blood counts, evolution of clonal/malignant disease, CSA side effects) |
| --- | --- | --- | --- | --- |
| Matsuda | 2002 | N = 10; SCT | Japan | Surrogate parameters (hemoglobin) |
| Goerner | 2002 | N = 405; SCT | USA | Surrogate parameters (GvHD, Infections, Karnofsky score and others) |
| Marsh | 1999 | N = 115; IST | Multinational | Surrogate paramters (Blood counts, time being free of transfusions, OS) |
| Deeg | 1998 | N = 212; SCT | USA | EORTC QLQ-C30 (plus self-developed BMT-module); DBMT; POMS; WHPQ |
| Passweg | 1997 | N = 1305; SCT | Multinational | Surrogate parameters (blood counts, GvHD, survival) |
| Andrykowski | 1995 | N = 1 (28)**; SCT  (** study comprised of 28 bmt patients including one with aplastic anemia) | USA | POMS, FLIC, PAIS, SIP |
| Novitzky | 1992 | N = 26; IST | South Africa | Surrogate parameters (return to work, blood counts) |
| Najean | 1990 | N = 156; failed SCT, IST (+ androgens) | France | Surrogate parameters (blood counts, occupation, marriage, pregnancies) |
| De Planque | 1989 | N = 468; IST | Multinational | Surrogate parameters (blood counts, survival, clonal evolution) |
| Hinterberger | 1987 | N = 23; SCT | Austria | Surrogate parameters (blood counts, GvHD) |
| Bayever | 1984 | N = 57; SCT, IST (pediatric pts. up to the age of 25) | USA | Surrogate parameters (Karnofsky score, GvHD) |

SCT = stem cell transplantation; IST = immunosuppressive therapy; BMT = bone marrow transplantation; SF36 = Medical Outcome Study Short Form 36 Health Survey; SCL90-R = Symptoms Checklist Revised; FRI = Family Relations Index; SSQSR = Social Support Questionnaire Short Form; NBRS **=** Neurobehavioral Rating Scale; MMQ = Modified Memory Questionnaire; OS = overall surival, GvHD = Graft versus Host Disease; Q-TWiST = quality-adjusted time without symptoms and toxicity; CSA = cyclosporine A; DBMT = Demands of BMT Recovery Inventory; POMS = Profile of Mood States; WHPQ = Ware Health Perceptions Questionnaire; FLIC = Functional Living Index – Cancer; PAIS = Psychological Adjustment to Illness Scale; SIP = Sickness Impact Profile

Table 2 Literature search: Studies reporting about quality of life in adult patients with paroxysmal nocturnal hemoglobinuria (PNH)

| **Author** | **Year** | **No. of patients evaluated; form of treatment (SCT/eculizumab/other)** | **Country of origin** | **QoL instrument used** |
| --- | --- | --- | --- | --- |
| Matos-Fernandez | 2009 | N = 117; SCT (review of SCT in PNH) | Multinational | Surrogate parameters (GvHD, survival) |
| Brodsky | 2008 | N = 93; eculizumab (vs placebo) | Multinational | EORTC QLQ-C30, FACIT |
| Meyers | 2007 | N = 29; other | Multinational | EORTC QLQ-C30, FACIT plus self-reported symptoms |
| Hillmen | 2006 | N = 87; eculizumab (vs placebo) | Multinational | EORTC QLQ-C30, FACIT |
| Hill | 2005 | N = 11 | UK | EORTC QLQ-C30 |
| Raiola | 2000 | N = 7; SCT | Italia | Surrogate parameters (Blood counts, GvHD) |

FACIT = Functional Assessment of Chronic Illness Therapy- Fatigue Scale, GvHD = Graft versus Host Disease

**Table 3** Patient characteristics of the patients interviewed during phase I and II

|  | Phase I (n = 19) | Phase II (n = 30) |
| --- | --- | --- |
| Sex  Female  Male | 15 (79%)  4 (21%) | 15 (50%)  15 (50%) |
| Disease*  PNH  AA  AA/PNH | 4 (21%)  9 (47%)  6 (32%) | 10 (33%)  10 (33%)  10 (33%) |
| Previous therapies*  BMT  CSA  ATG  eculizumab | 3 (16%)  13 (68%)  13 (68%)  6 (32%) | 2 (7%)  20 (67%)  17 (57%)  15 (53%) |
| Age at diagnosis (in years)*  Median; mean; range | 29.1; 30.7; 17.0 – 50.3 | 31.3; 43.2; 13.9 – 60.3 |
| Age at the time of interview (in years)  Median; mean; range | 42.1; 40.7; 25.1 – 61.3 | 43.7; 43.3; 18.9 – 73.5 |
| Time from first symptoms until first physician contact (in months)*  Median; mean; range | 0; 9; 0 - 56 | 0; 3; 0 - 24 |
| Time from first physician contact to final diagnosis (in months)*  Median; mean; range | 5; 14; 0 - 56 | 2; 6; 0 - 37 |

*self-reported; CSA = Cyclosporin A, BMT = bone marrow transplantation, ATG = Antithymocyte Globulin

**Table 4** Comparison of issues from phase I with items from the EORTC QLQ-C30 questionnaire

| EORTC QLQ-C30 items | Patient rating (mean; median) |
| --- | --- |
| Mentioned and rated important   1. Do you have any trouble doing strenuous activities, like carrying a heavy shopping bag or a suitcase? 2. Do you have any trouble taking a long walk? 3. Do you need to stay in bed or a chair during the day? 4. Were you limited in doing either your work or other daily activities? 5. Were you limited in pursuing your hobbies or other leisure time activities? 6. Were you short of breath? 7. Did you need to rest? 8. Have you had pain? 9. Did pain interfere with your daily activities? 10. Have you had trouble sleeping? 11. Have you felt weak? 12. Were you tired? 13. Have you had difficulty in concentrating on things, like reading a newspaper or watching television? 14. Did you feel tense? 15. Did you worry? 16. Did you feel depressed? | 3.2; 3.0    2.6; 3.0  2.3; 2.0  2.4; 2.0  2.4; 2.0  2.9; 3.0  3.3; 4.0  2.5; 3.0  2.3; 3.0  2.2; 2.0  2.6; 3.0  3.1; 3.0  2.4; 2.0  2.3; 2.0  2.7; 3.0  2.4; 2.0 |
| Mentioned and rated unimportant   1. Do you have any trouble taking a short walk outside of the house? 2. Do you need help with eating, dressing, washing yourself or using the toilet? 3. Have you felt nauseated? 4. Have you had diarrhea? 5. Has your physical condition or medical treatment caused you financial difficulties? 6. Has your physical condition or medical treatment interfered with your social activities? 7. Has your physical condition or medical treatment interfered with your family life? | 1.8; 1.0  1.2; 1.0  1.6; 1.0  1.5; 1.0  1.9; 1.0  1.7; 1.0  1.7; 1.0 |
| Not mentioned   1. Have you lacked appetite? 2. Have you vomited? 3. Have you been constipated? 4. Did you feel tense? 5. Have you had difficulty remembering things? | n.a.  n.a.  n.a.  n.a.  n.a. |

n.a. = not applicable

**References**

1. Gulbis B, Eleftheriou A, Angastiniotis M, Ball S, Surralles J, Castella M, Heimpel H, Hill A, Corrons JL (2010) Epidemiology of rare anaemias in Europe. Advances in experimental medicine and biology 686:375-396. doi:10.1007/978-90-481-9485-8_22

2. Passweg JR, Marsh JC (2010) Aplastic anemia: first-line treatment by immunosuppression and sibling marrow transplantation. Hematology / the Education Program of the American Society of Hematology American Society of Hematology Education Program 2010:36-42. doi:10.1182/asheducation-2010.1.36

3. Young NS (2002) Acquired aplastic anemia. Annals of internal medicine 136 (7):534-546

4. Parker CJ (2007) The pathophysiology of paroxysmal nocturnal hemoglobinuria. Experimental hematology 35 (4):523-533. doi:10.1016/j.exphem.2007.01.046

5. Hillmen P, Lewis SM, Bessler M, Luzzatto L, Dacie JV (1995) Natural history of paroxysmal nocturnal hemoglobinuria. N Engl J Med 333 (19):1253-1258. doi:10.1056/NEJM199511093331904

6. Socie G, Mary JY, de Gramont A, Rio B, Leporrier M, Rose C, Heudier P, Rochant H, Cahn JY, Gluckman E (1996) Paroxysmal nocturnal haemoglobinuria: long-term follow-up and prognostic factors. French Society of Haematology. Lancet 348 (9027):573-577

7. Moyo VM, Mukhina GL, Garrett ES, Brodsky RA (2004) Natural history of paroxysmal nocturnal haemoglobinuria using modern diagnostic assays. Br J Haematol 126 (1):133-138. doi:10.1111/j.1365-2141.2004.04992.x

8. Nishimura J, Kanakura Y, Ware RE, Shichishima T, Nakakuma H, Ninomiya H, Decastro CM, Hall S, Kanamaru A, Sullivan KM, Mizoguchi H, Omine M, Kinoshita T, Rosse WF (2004) Clinical course and flow cytometric analysis of paroxysmal nocturnal hemoglobinuria in the United States and Japan. Medicine (Baltimore) 83 (3):193-207

9. Hillmen P, Muus P, Roth A, Elebute MO, Risitano AM, Schrezenmeier H, Szer J, Browne P, Maciejewski JP, Schubert J, Urbano-Ispizua A, de Castro C, Socie G, Brodsky RA (2013) Long-term safety and efficacy of sustained eculizumab treatment in patients with paroxysmal nocturnal haemoglobinuria. Br J Haematol 162 (1):62-73. doi:10.1111/bjh.12347

10. Marsh JC, Kulasekararaj AG (2013) Management of the refractory aplastic anemia patient: what are the options? Blood 122 (22):3561-3567. doi:10.1182/blood-2013-05-498279

11. Hillmen P, Young NS, Schubert J, Brodsky RA, Socie G, Muus P, Roth A, Szer J, Elebute MO, Nakamura R, Browne P, Risitano AM, Hill A, Schrezenmeier H, Fu CL, Maciejewski J, Rollins SA, Mojcik CF, Rother RP, Luzzatto L (2006) The complement inhibitor eculizumab in paroxysmal nocturnal hemoglobinuria. N Engl J Med 355 (12):1233-1243. doi:10.1056/NEJMoa061648

12. Brodsky RA, Young NS, Antonioli E, Risitano AM, Schrezenmeier H, Schubert J, Gaya A, Coyle L, de Castro C, Fu CL, Maciejewski JP, Bessler M, Kroon HA, Rother RP, Hillmen P (2008) Multicenter phase 3 study of the complement inhibitor eculizumab for the treatment of patients with paroxysmal nocturnal hemoglobinuria. Blood 111 (4):1840-1847. doi:10.1182/blood-2007-06-094136

13. Fayers P, Bottomley A, Group EQoL, Quality of Life U (2002) Quality of life research within the EORTC-the EORTC QLQ-C30. European Organisation for Research and Treatment of Cancer. European journal of cancer 38 Suppl 4:S125-133

14. Meyers G, Weitz I, Lamy T, Cahn JY, Kroon HA, Severino B, Uranga MT, Alonso MS, Vela JAG, Hill A (2007) Disease-related symptoms reported across a broad population of patients with paroxysmal nocturnal hemoglobinuria. Blood 110 (11):1076A-1076A

15. Schrezenmeier H, Muus P, Socie G, Szer J, Urbano-Ispizua A, Maciejewski JP, Brodsky RA, Bessler M, Kanakura Y, Rosse W, Khursigara G, Bedrosian C, Hillmen P (2014) Baseline characteristics and disease burden in patients in the International Paroxysmal Nocturnal Hemoglobinuria Registry. Haematologica 99 (5):922-929. doi:10.3324/haematol.2013.093161

16. Curbow B, Somerfield MR, Baker F, Wingard JR, Legro MW (1993) Personal changes, dispositional optimism, and psychological adjustment to bone marrow transplantation. Journal of behavioral medicine 16 (5):423-443

17. Somerfield MR, Curbow B, Wingard JR, Baker F, Fogarty LA (1996) Coping with the physical and psychosocial sequelae of bone marrow transplantation among long-term survivors. Journal of behavioral medicine 19 (2):163-184

18. Eapen M, Ramsay NK, Mertens AC, Robison LL, DeFor T, Davies SM (2000) Late outcomes after bone marrow transplant for aplastic anaemia. Br J Haematol 111 (3):754-760

19. Passweg JR, Socie G, Hinterberger W, Bacigalupo A, Biggs JC, Camitta BM, Champlin RE, Gale RP, Gluckman E, Gordon-Smith EC, Hows JM, Klein JP, Nugent ML, Pasquini R, Rowlings PA, Speck B, Tichelli A, Zhang MJ, Horowitz MM, Bortin MM (1997) Bone marrow transplantation for severe aplastic anemia: has outcome improved? Blood 90 (2):858-864

20. Frickhofen N, Heimpel H, Kaltwasser JP, Schrezenmeier H, German Aplastic Anemia Study G (2003) Antithymocyte globulin with or without cyclosporin A: 11-year follow-up of a randomized trial comparing treatments of aplastic anemia. Blood 101 (4):1236-1242. doi:10.1182/blood-2002-04-1134

21. Scheinberg P, Nunez O, Weinstein B, Scheinberg P, Biancotto A, Wu CO, Young NS (2011) Horse versus rabbit antithymocyte globulin in acquired aplastic anemia. N Engl J Med 365 (5):430-438. doi:10.1056/NEJMoa1103975

22. de Planque MM, Bacigalupo A, Wursch A, Hows JM, Devergie A, Frickhofen N, Brand A, Nissen C (1989) Long-term follow-up of severe aplastic anaemia patients treated with antithymocyte globulin. Severe Aplastic Anaemia Working Party of the European Cooperative Group for Bone Marrow Transplantation (EBMT). Br J Haematol 73 (1):121-126

23. Najean Y, Haguenauer O (1990) Long-term (5 to 20 years) Evolution of nongrafted aplastic anemias. The Cooperative Group for the Study of Aplastic and Refractory Anemias. Blood 76 (11):2222-2228

24. Viollier R, Passweg J, Gregor M, Favre G, Kuhne T, Nissen C, Gratwohl A, Tichelli A (2005) Quality-adjusted survival analysis shows differences in outcome after immunosuppression or bone marrow transplantation in aplastic anemia. Annals of hematology 84 (1):47-55. doi:10.1007/s00277-004-0930-3

25. Johnson C, Aaronson N, Blazeby J, Bottomley A, Fayers P, Koller M, Kuliś D, Ramage J, Sprangers M, Velikova G, Young T (2011) Guidelines for Developing Questionnaire Modules.

EORTC Quality of Life Department, Ave. E. Mounier 83, B.11, 1200 Brussels, BELGIUM, Tel: +32 (0)2 774 1678

26. Singer S, Arraras JI, Baumann I, Boehm A, Chie WC, Galalae R, Langendijk JA, Guntinas-Lichius O, Hammerlid E, Pinto M, Nicolatou-Galitis O, Schmalz C, Sen M, Sherman AC, Spiegel K, Verdonck-de Leeuw I, Yarom N, Zotti P, Hofmeister D, Group EQoL, Head E, Neck Cancer G (2013) Quality of life in patients with head and neck cancer receiving targeted or multimodal therapy--update of the EORTC QLQ-H&N35, Phase I. Head & neck 35 (9):1331-1338. doi:10.1002/hed.23127

27. Duell T, van Lint MT, Ljungman P, Tichelli A, Socie G, Apperley JF, Weiss M, Cohen A, Nekolla E, Kolb HJ (1997) Health and functional status of long-term survivors of bone marrow transplantation. EBMT Working Party on Late Effects and EULEP Study Group on Late Effects. European Group for Blood and Marrow Transplantation. Annals of internal medicine 126 (3):184-192

28. Peinemann F, Labeit AM (2014) Stem cell transplantation of matched sibling donors compared with immunosuppressive therapy for acquired severe aplastic anaemia: a Cochrane systematic review. BMJ open 4 (7):e005039. doi:10.1136/bmjopen-2014-005039

29. Bayever E, Champlin R, Ho W, Lenarsky C, Storch S, Ladisch S, Gale RP, Feig SA (1984) Comparison between bone marrow transplantation and antithymocyte globulin in treatment of young patients with severe aplastic anemia. The Journal of pediatrics 105 (6):920-925

30. Hinterberger W, Gadner H, Hocker P, Hajek-Rosenmayr A, Graninger W, Grabner G, Volc-Platzer B, Hawliczek R, Karcher KH, Kallinger W, et al. (1987) Survival and quality of life in 23 patients with severe aplastic anemia treated with bone marrow transplantation (BMT). Blut 54 (3):137-146

31. Novitzky N, Mobara G, Jacobs P (1992) Antilymphocyte globulin and high-dose methylprednisolone improve survival in patients with aplastic anaemia without additional financial costs. South African medical journal = Suid-Afrikaanse tydskrif vir geneeskunde 81 (5):254-257

32. Marsh J, Schrezenmeier H, Marin P, Ilhan O, Ljungman P, McCann S, Socie G, Tichelli A, Passweg J, Hows J, Raghavachar A, Locasciulli A, Bacigalupo A (1999) Prospective randomized multicenter study comparing cyclosporin alone versus the combination of antithymocyte globulin and cyclosporin for treatment of patients with nonsevere aplastic anemia: a report from the European Blood and Marrow Transplant (EBMT) Severe Aplastic Anaemia Working Party. Blood 93 (7):2191-2195

33. Goerner M, Gooley T, Flowers ME, Sullivan KM, Kiem HP, Sanders JE, Martin PJ, Storb R (2002) Morbidity and mortality of chronic GVHD after hematopoietic stem cell transplantation from HLA-identical siblings for patients with aplastic or refractory anemias. Biology of blood and marrow transplantation : journal of the American Society for Blood and Marrow Transplantation 8 (1):47-56

34. Matsuda A, Kishimoto K, Yoshida K, Yagasaki F, Ito Y, Sakata T, Kawai N, Ino H, Hirashima K, Bessho M (2002) Long-term follow-up of patients with aplastic anemia and refractory anemia responding to combination therapy with recombinant human granulocyte colony-stimulating factor and erythropoietin. International journal of hematology 76 (3):244-250

35. Resnick IB, Aker M, Shapira MY, Tsirigotis PD, Bitan M, Abdul-Hai A, Samuel S, Ackerstein A, Gesundheit B, Zilberman I, Miron S, Yoffe L, Lvovich A, Slavin S, Or R (2006) Allogeneic stem cell transplantation for severe acquired aplastic anaemia using a fludarabine-based preparative regimen. Br J Haematol 133 (6):649-654. doi:10.1111/j.1365-2141.2006.06084.x

36. Gupta V, Eapen M, Brazauskas R, Carreras J, Aljurf M, Gale RP, Hale GA, Ilhan O, Passweg JR, Ringden O, Sabloff M, Schrezenmeier H, Socie G, Marsh JC (2010) Impact of age on outcomes after bone marrow transplantation for acquired aplastic anemia using HLA-matched sibling donors. Haematologica 95 (12):2119-2125. doi:10.3324/haematol.2010.026682

37. Hill A, Hillmen P, Richards SJ, Elebute D, Marsh JC, Chan J, Mojcik CF, Rother RP (2005) Sustained response and long-term safety of eculizumab in paroxysmal nocturnal hemoglobinuria. Blood 106 (7):2559-2565. doi:10.1182/blood-2005-02-0564

38. Raiola AM, Van Lint MT, Lamparelli T, Gualandi F, Benvenuto F, Figari O, Mordini N, Berisso G, Bregante S, Frassoni F, Bacigalupo A (2000) Bone marrow transplantation for paroxysmal nocturnal hemoglobinuria. Haematologica 85 (1):59-62

39. Matos-Fernandez NA, Abou Mourad YR, Caceres W, Kharfan-Dabaja MA (2009) Current status of allogeneic hematopoietic stem cell transplantation for paroxysmal nocturnal hemoglobinuria. Biology of blood and marrow transplantation : journal of the American Society for Blood and Marrow Transplantation 15 (6):656-661. doi:10.1016/j.bbmt.2008.12.507

40. Malvezzi M, Bertuccio P, Rosso T, Rota M, Levi F, La Vecchia C, Negri E (2015) European cancer mortality predictions for the year 2015: does lung cancer have the highest death rate in EU women? Annals of oncology : official journal of the European Society for Medical Oncology / ESMO 26 (4):779-786. doi:10.1093/annonc/mdv001

41. Schrezenmeier H, Hochsmann B (2012) Drugs that inhibit complement. Transfusion and apheresis science : official journal of the World Apheresis Association : official journal of the European Society for Haemapheresis 46 (1):87-92. doi:10.1016/j.transci.2011.11.012

42. Sanders JE, Hoffmeister PA, Storer BE, Appelbaum FR, Storb RF, Syrjala KL (2010) The quality of life of adult survivors of childhood hematopoietic cell transplant. Bone Marrow Transplant 45 (4):746-754. doi:10.1038/bmt.2009.224

43. Sanders JE, Woolfrey AE, Carpenter PA, Storer BE, Hoffmeister PA, Deeg HJ, Flowers ME, Storb RF (2011) Late effects among pediatric patients followed for nearly 4 decades after transplantation for severe aplastic anemia. Blood 118 (5):1421-1428. doi:10.1182/blood-2011-02-334953

44. Frickhofen N, Kaltwasser JP, Schrezenmeier H, Raghavachar A, Vogt HG, Herrmann F, Freund M, Meusers P, Salama A, Heimpel H (1991) Treatment of aplastic anemia with antilymphocyte globulin and methylprednisolone with or without cyclosporine. The German Aplastic Anemia Study Group. N Engl J Med 324 (19):1297-1304. doi:10.1056/NEJM199105093241901

45. Deeg HJ, Leisenring W, Storb R, Nims J, Flowers ME, Witherspoon RP, Sanders J, Sullivan KM (1998) Long-term outcome after marrow transplantation for severe aplastic anemia. Blood 91 (10):3637-3645

46. Andrykowski MA, Bruehl S, Brady MJ, Henslee-Downey PJ (1995) Physical and psychosocial status of adults one-year after bone marrow transplantation: a prospective study. Bone Marrow Transplant 15 (6):837-844

**Supplemental Tables 5.1 and 5.2**

The following table shows the 141 Issues concerning the patients’ quality of life except for the issues concerning health care problems. Shown are the number of fulfilled relevance criteria, the mean and median ranking of importance for all patients (n=30), and the median ranking for patients with PNH (n=10), for patients with AA (N=10) and for patients with AA/PNH (n=10). Issues, rated ≥ 2 points higher (median) from AA-patients compared to PNH-patients are highlighted in red (I10); issues, rated ≥ 2 points higher (median) from PNH-patients compared to AA-patients are highlighted in blue (I67, I70).

|  |  | **Number of relevant criteria fulfilled** | **Mean (all pts)** | **Median (all pts)** | **Median PNH pts** | **Median AA pts** | **Median PNH/AA pts** |
| --- | --- | --- | --- | --- | --- | --- | --- |
| **I1** | Fatigue | 4,0 | 3,3 | 3,0 | 3,0 | 4,0 | 3,0 |
| **I2** | Normal rhythm of life affected | 4,0 | 3,0 | 3,0 | 3,0 | 2,5 | 3,5 |
| **I3** | Not being healthy | 4,0 | 3,0 | 3,0 | 3,5 | 2,5 | 3,5 |
| **I4** | Dyspnea/shortage of breath | 4,0 | 2,9 | 3,0 | 3,0 | 3,5 | 3,0 |
| **I5** | Uncertain future | 4,0 | 2,9 | 3,0 | 3,5 | 3,0 | 3,0 |
| **I6** | Having to get on with the disease forever/lifelong | 4,0 | 2,9 | 3,0 | 3,5 | 3,0 | 3,0 |
| **I7** | I can’t accomplish what I’d like to do | 4,0 | 2,9 | 3,0 | 3,5 | 3,0 | 3,5 |
| **I8** | Not being able to be flexible | 4,0 | 2,7 | 3,0 | 2,5 | 1,5 | 3,5 |
| **I9** | Disease as center of life/ everything has to move around the disease | 4,0 | 2,7 | 3,0 | 2,0 | 3,0 | 3,0 |
| **I10** | Fear of therapy failure | 4,0 | 2,7 | 3,0 | 1,5 | 4,0 | 3,0 |
| **I11** | Not being able to manage the daily routine/everyday life by myself | 4,0 | 2,4 | 2,0 | 2,0 | 3,0 | 2,5 |
| **I12** | Trouble concentrating | 4,0 | 2,4 | 2,0 | 2,0 | 2,0 | 3,0 |
| **I13** | No energy/power to engage in private life/hobbies | 4,0 | 2,4 | 2,0 | 2,0 | 3,0 | 2,0 |
|  |  | **Number of relevant criteria fulfilled** | **Mean (all pts)** | **Median (all pts)** | **Median PNH pts** | **Median AA pts** | **Median PNH/AA pts** |
| **I14** | Fear of bone marrow transplantation | 4,0 | 2,3 | 2,0 | 1,0 | 2,0 | 1,5 |
| **I15** | Support of family and friends is helping | 3,0 | 3,7 | 4,0 | 4,0 | 4,0 | 4,0 |
| **I16** | To be treated „normal“ | 3,0 | 3,6 | 4,0 | 4,0 | 4,0 | 4,0 |
| **I17** | I have to pause/relax more often than I’d like to | 3,0 | 3,3 | 4,0 | 3,5 | 4,0 | 3,0 |
| **I18** | Trouble walking stairs | 3,0 | 3,2 | 3,0 | 3,0 | 4,0 | 3,0 |
| **I19** | One hast to be careful not to catch an infection | 3,0 | 3,1 | 4,0 | 4,0 | 3,0 | 4,0 |
| **I20** | Being tired (despite sleep) | 3,0 | 3,1 | 3,0 | 3,0 | 3,5 | 3,5 |
| **I21** | Fear of changing blood counts | 3,0 | 3,1 | 3,0 | 2,5 | 3,5 | 4,0 |
| **I22** | Listlessness | 3,0 | 2,9 | 3,0 | 2,0 | 3,5 | 3,0 |
| **I23** | Fear of relapse or disease progress | 3,0 | 2,8 | 3,0 | 2,5 | 3,5 | 3,0 |
| **I24** | Vacation/holidays/travel only possible within certain limits | 3,0 | 2,8 | 3,0 | 3,0 | 2,0 | 3,0 |
| **I25** | Blood counts dictate/determine mood | 3,0 | 2,8 | 3,0 | 2,0 | 3,0 | 3,0 |
| **I26** | It helps to exchange thoughts/fears/experiences with other patients with PNH and/or AA | 3,0 | 2,8 | 3,0 | 3,5 | 3,5 | 2,5 |
|  |  | **Number of relevant criteria fulfilled** | **Mean (all pts)** | **Median (all pts)** | **Median PNH pts** | **Median AA pts** | **Median PNH/AA pts** |
| **I27** | Having too many worries | 3,0 | 2,7 | 3,0 | 3,0 | 2,5 | 3,0 |
| **I28** | Loss of lightheartedness | 3,0 | 2,7 | 3,0 | 2,5 | 3,5 | 2,5 |
| **I29** | Problems within work routine/studies/at school | 3,0 | 2,7 | 3,0 | 2,5 | 2,5 | 3,0 |
| **I30** | Troubles to keep balance between excessive demands/ being overworked and under challenged | 3,0 | 2,6 | 3,0 | 1,5 | 3,0 | 3,0 |
| **I31** | Pain within muscles and joints | 3,0 | 2,5 | 3,0 | 2,5 | 2,5 | 3,0 |
| **I32** | Infections | 3,0 | 2,5 | 2,0 | 2,0 | 2,0 | 3,0 |
| **I33** | Increased chance of bleeding | 3,0 | 2,4 | 2,0 | 1,0 | 2,5 | 2,5 |
| **I34** | Not being able to cope with demands at work/studies/school | 3,0 | 2,3 | 2,0 | 2,5 | 2,0 | 2,0 |
| **I35** | Fear that there will be no more treatment left for me | 3,0 | 2,1 | 2,0 | 1,5 | 1,0 | 3,0 |
| **I36** | Proud about one’s accomplishments | 2,0 | 3,1 | 3,0 | 3,0 | 3,5 | 3,5 |
| **I37** | To be faced/confronted with the disease again and again | 2,0 | 3,0 | 3,0 | 2,5 | 3,0 | 3,5 |
| **I38** | Sympathy from my surroundings helps | 2,0 | 2,9 | 3,0 | 3,0 | 3,0 | 3,0 |
| **I39** | It helps being able to be flexible at work/studies/school | 2,0 | 2,8 | 3,0 | 2,0 | 3,0 | 4,0 |
|  |  | **Number of relevant criteria fulfilled** | **Mean (all pts)** | **Median (all pts)** | **Median PNH pts** | **Median AA pts** | **Median PNH/AA pts** |
| **I40** | One has to be alert for even the most minor/smallest because they could mean serious trouble | 2,0 | 2,8 | 3,0 | 3,0 | 3,0 | 3,0 |
| **I41** | Future prospects are limited | 2,0 | 2,8 | 3,0 | 2,0 | 2,5 | 3,0 |
| **I42** | I am stressed because I sense that my surroundings are stressed | 2,0 | 2,8 | 3,0 | 3,0 | 3,0 | 3,0 |
| **I43** | Sympathy and respect at work/studies/school helps | 2,0 | 2,8 | 3,0 | 3,0 | 2,5 | 3,5 |
| **I44** | Trouble jogging/running (5km) /doing sports | 2,0 | 2,7 | 3,0 | 2,5 | 4,0 | 3,5 |
| **I45** | Not being able to plan ahead | 2,0 | 2,7 | 3,0 | 2,0 | 3,0 | 3,0 |
| **I46** | Thoughts center around one theme/thinking about the disease the whole time | 2,0 | 2,7 | 3,0 | 2,0 | 3,0 | 2,5 |
| **I47** | Resignation from certain sports | 2,0 | 2,6 | 3,0 | 2,5 | 2,5 | 3,0 |
| **I48** | Losing control of my own body | 2,0 | 2,6 | 2,0 | 2,0 | 2,5 | 3,0 |
| **I49** | Trouble walking more up to 2km or longer walks | 2,0 | 2,6 | 3,0 | 2,0 | 3,5 | 3,0 |
| **I50** | Body feels heavy | 2,0 | 2,6 | 3,0 | 3,0 | 3,0 | 3,0 |
| **I51** | Trouble to portion my strength/power | 2,0 | 2,6 | 3,0 | 2,5 | 3,0 | 3,0 |
| **I52** | The need to be careful | 2,0 | 2,6 | 3,0 | 2,0 | 3,0 | 3,0 |
|  |  | **Number of relevant criteria fulfilled** | **Mean (all pts)** | **Median (all pts)** | **Median PNH pts** | **Median AA pts** | **Median PNH/AA pts** |
| **I53** | Being exhausted for days on end after exertion/strenuous efforts | 2,0 | 2,5 | 3,0 | 3,0 | 2,5 | 3,0 |
| **I54** | Not being able to be spontaneous | 2,0 | 2,5 | 2,0 | 2,0 | 1,5 | 3,0 |
| **I55** | One always loses track/is thrown off course | 2,0 | 2,5 | 3,0 | 2,5 | 1,5 | 3,0 |
| **I56** | Visible symptoms remind me all the time that I am sick | 2,0 | 2,5 | 2,0 | 2,5 | 1,5 | 3,0 |
| **I57** | Precautions because of the risk of infections | 2,0 | 2,5 | 2,0 | 2,0 | 2,5 | 3,0 |
| **I58** | To miss something in life | 2,0 | 2,4 | 2,0 | 2,0 | 1,5 | 3,0 |
| **I59** | Feeling downhearted | 2,0 | 2,4 | 2,0 | 2,5 | 2,0 | 2,5 |
| **I60** | Having trouble to stand | 2,0 | 2,4 | 2,0 | 1,5 | 2,5 | 3,0 |
| **I61** | To feel helpless in life | 2,0 | 2,4 | 2,0 | 2,5 | 2,0 | 2,0 |
| **I62** | Dizziness | 2,0 | 2,3 | 2,0 | 2,0 | 2,5 | 2,5 |
| **I63** | To feel vulnerable | 2,0 | 2,3 | 2,0 | 2,0 | 2,5 | 3,0 |
| **I64** | Have the feeling of not moving forward | 2,0 | 2,3 | 2,0 | 2,0 | 2,0 | 3,0 |
| **I65** | Crankiness/irritability | 2,0 | 2,3 | 2,0 | 2,0 | 2,0 | 2,0 |
| **I66** | Having trouble getting out of bed in the morning | 2,0 | 2,3 | 2,0 | 2,0 | 2,5 | 2,0 |
| **I67** | Appearance | 2,0 | 2,2 | 2,0 | 3,0 | 1,0 | 3,0 |
|  |  | **Number of relevant criteria fulfilled** | **Mean (all pts)** | **Median (all pts)** | **Median PNH pts** | **Median AA pts** | **Median PNH/AA pts** |
| **I68** | Uneasiness | 2,0 | 2,2 | 2,0 | 2,0 | 2,5 | 1,5 |
| **I69** | No flexibility in the planning of studies/schooling/education/job | 2,0 | 2,2 | 2,0 | 1,5 | 1,5 | 3,0 |
| **I70** | Sleep disturbance | 2,0 | 2,2 | 2,0 | 3,0 | 1,0 | 2,0 |
| **I71** | I have to be reasonable | 2,0 | 2,1 | 2,0 | 2,0 | 1,5 | 2,5 |
| **I72** | Not being able to pull oneself together | 2,0 | 2,1 | 2,0 | 2,0 | 2,0 | 2,5 |
| **I73** | Swellings/inflammation within the mouth/sore mouth | 2,0 | 2,1 | 2,0 | 2,0 | 2,5 | 1,0 |
| **I74** | To like it being within ones body | 2,0 | 2,1 | 2,0 | 1,0 | 1,0 | 2,5 |
| **I75** | Problems getting the household/-work done | 2,0 | 2,0 | 2,0 | 2,5 | 1,0 | 2,5 |
| **I76** | To become upset because one has to explain oneself all the time | 2,0 | 2,0 | 2,0 | 1,5 | 1,5 | 2,5 |
| **I77** | *For men:* erectile dysfunction | 2,0 | 2,0 | 2,0 | 2,0 | 1,0 | 2,0 |
| All issues shown above met at least 2 of the defined criteria, so that they were regarded as important. **Issues I78 to I141 met one or less criteria they therefore were regarded as being unimportant.** | | | | | | | |

|  |  | **Number of relevant criteria fulfilled** | **Mean (all pts)** | **Median (all pts)** | **Median PNH pts** | **Median AA pts** | **Median PNH/AA pts** |
| --- | --- | --- | --- | --- | --- | --- | --- |
| **I78** | Bruises | 1,0 | 2,2 | 2,0 | 2,0 | 2,0 | 2,5 |
| **I79** | Less activities with friends | 1,0 | 2,2 | 2,0 | 2,0 | 2,0 | 2,0 |
| **I80** | Having to quit work/studies/school; not being able to go to, becoming redundant | 1,0 | 2,2 | 1,0 | 2,0 | 2,0 | 1,0 |
| **I81** | Limited self-determination | 1,0 | 2,2 | 2,0 | 2,0 | 2,0 | 2,0 |
| **I82** | Trouble finding insurance company  (e.g. long-term disability) | 1,0 | 2,2 | 1,0 | 2,0 | 1,0 | 1,0 |
| **I83** | Trouble finding adequate sportive activities | 1,0 | 2,1 | 2,0 | 2,0 | 1,0 | 2,5 |
| **I84** | Gain of weight | 1,0 | 2,1 | 2,0 | 2,0 | 2,0 | 2,5 |
| **I85** | Fear to die | 1,0 | 2,1 | 2,0 | 1,5 | 2,0 | 2,0 |
| **I86** | Troubles to adjust conditions at work/at studies/at school according to my disease | 1,0 | 2,1 | 2,0 | 2,0 | 1,0 | 2,0 |
| **I87** | Unsure about what is allowed to do and what not | 1,0 | 2,1 | 2,0 | 2,0 | 2,0 | 2,0 |
| **I88** | Skin changes | 1,0 | 2,1 | 2,0 | 2,5 | 1,5 | 1,5 |
| **I89** | Headaches | 1,0 | 2,1 | 2,0 | 2,5 | 1,0 | 2,0 |
| **I90** | Back pain | 1,0 | 2,1 | 2,0 | 2,5 | 2,0 | 1,5 |
|  |  | **Number of relevant criteria fulfilled** | **Mean (all pts)** | **Median (all pts)** | **Median PNH pts** | **Median AA pts** | **Median PNH/AA pts** |
| **I91** | Great self-responsibility | 1,0 | 2,1 | 2,0 | 2,0 | 1,0 | 2,0 |
| **I92** | To always hover between life and death (feeling the sword of Damocles) | 1,0 | 2,0 | 2,0 | 1,0 | 2,0 | 2,0 |
| **I93** | (Spasmodic) abdominal pain | 1,0 | 2,0 | 2,0 | 2,0 | 1,5 | 2,0 |
| **I94** | I don’t have any chance to relax | 1,0 | 2,0 | 2,0 | 1,5 | 1,5 | 2,0 |
| **I95** | Discrepancy between the perception of the disease and the need to treat is stressful | 1,0 | 2,0 | 1,0 | 2,5 | 1,0 | 1,5 |
| **I96** | Anxiety because of the uncertainness where the disease comes from | 1,0 | 2,0 | 2,0 | 1,0 | 2,0 | 1,5 |
| **I97** | Speechlessness of relatives and friends | 1,0 | 2,0 | 2,0 | 1,0 | 2,5 | 2,0 |
| **I98** | Financial worries | 1,0 | 1,9 | 1,0 | 2,0 | 2,0 | 1,0 |
| **I99** | Can’t fulfill societies expectations | 1,0 | 1,8 | 1,0 | 1,0 | 1,0 | 3,0 |
| **I100** | Not interested in sexual activities | 1,0 | 1,7 | 1,0 | 1,5 | 1,0 | 2,0 |
| **I101** | *For women:* Loss of femininity | 1,0 | 1,7 | 1,0 | 1,0 | 1,0 | 2,0 |
| **I102** | Trembling | 0,0 | 1,9 | 2,0 | 2,0 | 2,0 | 1,5 |
| **I103** | Impaired Wound healing | 0,0 | 1,9 | 1,0 | 1,5 | 1,5 | 1,5 |
| **I104** | Sex is tiresome | 0,0 | 1,9 | 1,0 | 1,5 | 1,0 | 1,0 |
|  |  | **Number of relevant criteria fulfilled** | **Mean (all pts)** | **Median (all pts)** | **Median PNH pts** | **Median AA pts** | **Median PNH/AA pts** |
| **I105** | Being socially isolated/ feeling lonely | 0,0 | 1,9 | 1,0 | 1,0 | 2,0 | 1,5 |
| **I106** | Not being mobile | 0,0 | 1,9 | 1,0 | 1,0 | 1,5 | 2,0 |
| **I107** | Trouble walking short distances (500m) | 0,0 | 1,8 | 1,0 | 1,0 | 2,0 | 1,0 |
| **I108** | Troubles/worries of other people/friends/relatives make it difficult to live a normal life | 0,0 | 1,8 | 2,0 | 1,0 | 1,5 | 2,0 |
| **I109** | Having no one able to understand oneself | 0,0 | 1,8 | 1,0 | 1,0 | 2,0 | 1,0 |
| **I110** | I have to take care of each an every thing | 0,0 | 1,8 | 1,0 | 1,5 | 1,0 | 1,5 |
| **I111** | Not being satisfied with the picture others have about oneself | 0,0 | 1,8 | 2,0 | 1,0 | 1,5 | 2,0 |
| **I112** | Being exotic/an outcast of society | 0,0 | 1,8 | 1,0 | 1,0 | 1,0 | 2,0 |
| **I113** | Being useless | 0,0 | 1,8 | 1,0 | 1,0 | 1,0 | 2,0 |
| **I114** | Not being able to live with the disease | 0,0 | 1,8 | 1,0 | 1,5 | 1,0 | 2,0 |
| **I115** | To be unfree/limited regarding family planning | 0,0 | 1,8 | 1,0 | 1,0 | 1,0 | 1,0 |
| **I116** | Lack of understanding from my surroundings | 0,0 | 1,7 | 1,0 | 1,0 | 1,5 | 2,0 |
| **I117** | To totter/wiggle while walking | 0,0 | 1,7 | 1,0 | 1,0 | 1,5 | 2,0 |
| **I118** | Loss of self esteem | 0,0 | 1,7 | 1,0 | 1,5 | 1,0 | 2,0 |
| **I119** | Comparison with other patients with PNH, AA (e.g.. grudge, envy, fear, anxiety) | 0,0 | 1,7 | 1,0 | 2,0 | 1,0 | 1,0 |
|  |  | **Number of relevant criteria fulfilled** | **Mean (all pts)** | **Median (all pts)** | **Median PNH pts** | **Median AA pts** | **Median PNH/AA pts** |
| **I120** | Friends withdraw | 0,0 | 1,7 | 1,0 | 1,0 | 2,0 | 2,0 |
| **I121** | No/minimal interest of my surroundings in my disease | 0,0 | 1,7 | 1,0 | 1,0 | 1,5 | 1,5 |
| **I122** | I feel alone because I have trouble explaining my disease to others | 0,0 | 1,7 | 1,0 | 1,0 | 1,0 | 1,0 |
| **I123** | Problems in my relationship | 0,0 | 1,7 | 1,0 | 1,5 | 1,5 | 1,0 |
| **I124** | Feeling old | 0,0 | 1,7 | 1,0 | 1,0 | 1,0 | 2,0 |
| **I125** | Trouble finding work | 0,0 | 1,7 | 1,0 | 1,0 | 1,0 | 1,0 |
| **I126** | Nausea | 0,0 | 1,6 | 1,0 | 1,5 | 1,0 | 1,5 |
| **I127** | Fever | 0,0 | 1,6 | 1,0 | 1,0 | 1,0 | 1,5 |
| **I128** | Excessive demands through own children | 0,0 | 1,6 | 1,0 | 1,5 | 1,0 | 1,0 |
| **I129** | Having to hide the disease | 0,0 | 1,6 | 1,0 | 1,0 | 1,0 | 1,5 |
| **I130** | Stiff joints | 0,0 | 1,6 | 1,0 | 1,5 | 1,0 | 1,0 |
| **I131** | Discomfort swallowing/esophageal spasms | 0,0 | 1,5 | 1,0 | 1,5 | 1,0 | 1,0 |
| **I132** | Self-reproaches to be responsible getting the disease | 0,0 | 1,5 | 1,0 | 1,0 | 1,0 | 1,0 |
| **I133** | Others get the wrong/false impression of myself because they know nothing about the disease | 0,0 | 1,5 | 1,0 | 1,0 | 1,0 | 1,0 |
|  |  | **Number of relevant criteria fulfilled** | **Mean (all pts)** | **Median (all pts)** | **Median PNH pts** | **Median AA pts** | **Median PNH/AA pts** |
| **I134** | Diarrhea | 0,0 | 1,5 | 1,0 | 1,5 | 1,0 | 1,0 |
| **I135** | Feeling stigmatized | 0,0 | 1,5 | 1,0 | 1,0 | 1,0 | 2,0 |
| **I136** | Resignation/giving up | 0,0 | 1,5 | 1,0 | 1,0 | 1,0 | 1,0 |
| **I137** | Partner does not show emotions | 0,0 | 1,4 | 1,0 | 1,0 | 1,0 | 1,0 |
| **I138** | Sex has to be planed | 0,0 | 1,3 | 1,0 | 1,0 | 1,0 | 1,0 |
| **I139** | Adjustments of the living area | 0,0 | 1,3 | 1,0 | 1,0 | 1,0 | 1,0 |
| **I140** | Personal hygiene is hard for me | 0,0 | 1,2 | 1,0 | 1,0 | 1,0 | 1,0 |
| **I141** | Problems finding a partner | 0,0 | 1,1 | 1,0 | 1,0 | 1,0 | 1,0 |

##### Table 5.2

The following table shows the 34 Issues concerning the patients’ quality of life in regards to health care. Shown are the number of fulfilled relevance criteria, the mean and median ranking of importance for all patients (n=30), and the median ranking for patients with PNH (n=10), for patients with AA (N=10) and for patients with AA/PNH (n=10). Issues rated ≥ 2 points higher (median) from PNH-patients compared to AA-patients are highlighted in blue (I6, I7, I8, I20).

|  |  | **Number of relevant criteria fulfilled** | **Mean (all pts)** | **Median (all pts)** | **Median PNH pts** | **Median AA pts** | **Median PNH/AA pts** |
| --- | --- | --- | --- | --- | --- | --- | --- |
| **I1*** | Dependency on medical therapies (permanent, constantly) | 4,0 | 3,0 | 3,0 | 3,5 | 2,5 | 3,0 |
| **I2*** | It helps that I can trust my doctor/physician | 3,0 | 3,8 | 4,0 | 4,0 | 4,0 | 4,0 |
| **I3*** | It helps that my doctor/physician is insightful/perceptive/epathic | 3,0 | 3,5 | 4,0 | 4,0 | 3,0 | 4,0 |
| **I4*** | It helps being able to co-decide | 3,0 | 3,4 | 4,0 | 3,5 | 4,0 | 4,0 |
| **I5*** | It helps that I can reach my doctor/physician during a crisis | 3,0 | 3,4 | 4,0 | 4,0 | 3,5 | 4,0 |
| **I6*** | It helps to have a strong personal relationship with my doctor/physician (individual care) | 3,0 | 3,2 | 3,0 | 4,0 | 2,0 | 4,0 |
| **I7*** | It’s good that my doctors talk to each other/agree on my treatment (physician network, e.g. GP and hematologist) | 3,0 | 3,0 | 4,0 | 4,0 | 1,5 | 4,0 |
| **I8*** | Second opinion | 3,0 | 2,9 | 3,0 | 4,0 | 2,0 | 3,0 |
| **I9*** | Frequent confrontation with cancer patients and death | 3,0 | 2,3 | 2,0 | 2,0 | 3,0 | 2,0 |
| **I10*** | Uncertainty regarding therapeutic decision is quite wearing/stressing | 3,0 | 2,6 | 2,0 | 3,5 | 3,0 | 2,0 |
| **I11*** | Stressing/wearing therapeutic decisions | 3,0 | 2,4 | 2,0 | 2,5 | 2,0 | 2,0 |
| **I12*** | Examinations, therapies, doctor’s visits all take time | 2,0 | 2,7 | 3,0 | 3,0 | 2,0 | 3,0 |
|  |  | **Number of relevant criteria fulfilled** | **Mean (all pts)** | **Median (all pts)** | **Median PNH pts** | **Median AA pts** | **Median PNH/AA pts** |
| **I13*** | Medical team/personal does not know a lot about the disease | 2,0 | 2,5 | 2,0 | 3,5 | 2,0 | 2,0 |
| **I14*** | Treatment is unpleasant | 2,0 | 2,4 | 2,0 | 2,0 | 3,0 | 3,0 |
| **I15*** | Constant physician appointments interfere with work/job/studies/school | 2,0 | 2,3 | 2,0 | 2,5 | 2,0 | 3,0 |
| **I16*** | Dependency on medical team/caregivers | 2,0 | 2,3 | 2,0 | 2,5 | 2,0 | 2,0 |
| **I17*** | Doctor fixated on blood counts even though they do not reflect how I really feel | 2,0 | 2,2 | 2,0 | 2,0 | 3,0 | 2,5 |
| **I18*** | Psychological care/support | 2,0 | 2,2 | 2,0 | 2,0 | 1,0 | 2,0 |
| **I19*** | It is a problem, that the doctors are quite far away | 2,0 | 2,0 | 2,0 | 2,5 | 1,0 | 1,5 |
| **I20*** | Social legislation discrimination (approval of disability, health insurance problems, travel expenses etc.) | 2,0 | 2,0 | 1,0 | 3,0 | 1,0 | 1,0 |
| All issues shown in the table above met at least 2 of the defined criteria, so that they were regarded as important. **Issues I21 to I34 met one or less criteria, therefore they were regarded as being unimportant.** | | | | | | | |

|  |  | **Number of relevant criteria fulfilled** | **Mean (all pts)** | **Median (all pts)** | **Median PNH pts** | **Median AA pts** | **Median PNH/AA pts** |
| --- | --- | --- | --- | --- | --- | --- | --- |
| **I21*** | Trouble finding a good physician/doctor | 1,0 | 2,2 | 1,0 | 1,5 | 1,0 | 2,0 |
| **I22*** | Medical team/caregivers do not take enough time | 1,0 | 2,1 | 2,0 | 2,5 | 2,0 | 2,0 |
| **I23*** | I often have to explain facts to the doctors/physicians; I have to know more than my doctors/physicians | 1,0 | 2,0 | 2,0 | 2,0 | 1,0 | 2,5 |
| **I24*** | Elaborate organization of appointments is stressful | 1,0 | 2,0 | 2,0 | 2,0 | 1,5 | 2,0 |
| **I25*** | I am insufficiently informed about the disease | 1,0 | 2,0 | 2,0 | 2,0 | 1,5 | 1,0 |
| **I26*** | Fear that therapy will not be reimbursed | 1,0 | 1,9 | 1,0 | 1,5 | 1,0 | 2,5 |
| **I27*** | Feeling too much restricted because of physicians/doctors’ orders | 0,0 | 1,8 | 2,0 | 2,0 | 2,0 | 2,0 |
| **I28*** | To be left out in the rain with the diagnosis | 0,0 | 1,8 | 2,0 | 2,0 | 1,0 | 2,0 |
| **I29*** | Not being taken serious by caregivers | 0,0 | 1,8 | 1,0 | 1,5 | 1,5 | 1,5 |
| **I30*** | Conflicts with caregivers | 0,0 | 1,7 | 1,0 | 1,0 | 2,0 | 1,0 |
| **I31*** | Physicians do not agree on diagnosis/treatment etc. | 0,0 | 1,7 | 1,0 | 2,0 | 1,0 | 1,0 |
| **I32*** | Trouble getting adequate care (rehabilitation, reimbursement etc.) | 0,0 | 1,7 | 1,0 | 1,5 | 1,0 | 1,0 |
| **I33*** | Fear of losing doctor | 0,0 | 1,6 | 1,0 | 1,5 | 1,0 | 1,5 |
| **I34*** | Conflicts with health insurance | 0,0 | 1,6 | 1,0 | 1,0 | 1,0 | 1,0 |
